# Multidimensional analysis suggests that ZNF433 is a promising biomarker for the diagnosis and prognosis of human cancers

**DOI:** 10.3389/fonc.2025.1584042

**Published:** 2025-06-25

**Authors:** Lingyu Xie, Xin Zeng, Hui Luo, Bin Xie, Xuefeng Wang, Nan Wu, Junrong Zou, Guoxi Zhang, Xiaofeng Zou, Hui Xu

**Affiliations:** ^1^ The First Clinical College, Gannan Medical University, Ganzhou, Jiangxi, China; ^2^ Department of Urology, The First Affiliated Hospital of Gannan Medical University, Ganzhou, Jiangxi, China; ^3^ Department of Urology, Shanggao County People’s Hospital, Yichun, China; ^4^ Department of Surgery, Changshan County People’s Hospital, Quzhou, China; ^5^ Institute of Urology, The First Affiliated Hospital of Gannan Medical University, Ganzhou, Jiangxi, China; ^6^ Department of Urology, Jiangxi Engineering Technology Research Center of Calculi Prevention, Ganzhou, Jiangxi, China

**Keywords:** ZNF433, prognosis, diagnosis, biomarkers, bioinformatics

## Abstract

**Background:**

Zinc finger proteins, particularly members of the Krüppel-associated box zinc finger proteins (KRAB-ZFPs), play critical roles in regulating gene expression, cell cycle progression, and epigenetic modifications. Despite the growing body of research on KRAB-ZFPs, the role of ZNF433, a relatively less studied member of this family, remains poorly understood in the context of cancer.

**Methods:**

We conducted multi-dimensional analyses using publicly available databases, including TCGA and GTEx, to evaluate ZNF433’s expression patterns, genetic mutations, survival outcomes, immune microenvironment interactions, and diagnostic potential across different cancers. Functional enrichment and protein interaction network analyses were also performed to explore its potential involvement in cancer-related pathways.

**Results:**

Our findings revealed that ZNF433 is significantly downregulated in most cancer types, with stage-dependent expression patterns observed in KIRC and KIRP. High expression of ZNF433 was associated with improved overall survival (OS) in HNSC and KIRC, while in ESCA and PRAD, it was correlated with poorer disease-free survival (DFS). Additionally, high ZNF433 levels were linked to better DFS in BRCA, KIRP, THYM, and KIRC. ZNF433 expression was also closely associated with genomic instability markers, including tumor mutational burden (TMB), microsatellite instability (MSI), and mismatch repair (MMR) deficiencies. Furthermore, ZNF433 exhibited significant regulatory roles within the tumor immune microenvironment. Diagnostic analysis showed that ZNF433 has strong diagnostic potential in LAML and TGCT, and moderate diagnostic value in other cancers.

**Conclusions:**

Our study highlights the potential of ZNF433 as a diagnostic and prognostic biomarker and provides new insights into its potential as a therapeutic target.

## 1 Introduction

Zinc finger proteins are one of the largest transcription factor families in the human genome, capable of specifically recognizing DNA sequences and regulating gene expression. They play crucial roles in various biological processes, including development, cell differentiation, and tumorigenesis (1, 2).

KRAB-ZFPs are one of the largest subfamilies within the zinc finger protein family. They are distinguished by two functional domains: the N-terminal KRAB domain, which mediates transcriptional repression through chromatin remodeling, and the C-terminal zinc finger motif, which enables sequence-specific DNA recognition. These structural features facilitate their crucial role in regulating gene expression by recruiting co-repressor complexes (3, 4).

Previous studies have gradually revealed the crucial role of KRAB-ZFPs in tumorigenesis and progression, highlighting their significant functions in various malignancies (5–8). For instance, ZNF471 acts as a tumor suppressor in renal cell carcinoma by inhibiting the PI3K/AKT/mTOR signaling pathway, thereby reducing cell proliferation, migration, and inducing apoptosis (9). In breast cancer, ZNF57 functions as a tumor suppressor by regulating MEST expression to inhibit the Wnt/β-catenin signaling pathway, thereby affecting tumor cell proliferation (10). ZNF382 also serves as a tumor suppressor in gastric cancer by inhibiting cell proliferation, migration, and inducing apoptosis (11).Although research on KRAB-ZFPs in tumor biology has deepened and they have shown potential applications in cancer diagnosis and prognosis evaluation, studies on the pan-cancer roles of KRAB-ZFP members remain relatively limited (12–15).

ZNF433 is a member of the KRAB-ZFPs family, located on human chromosome 19, and encodes a transcription factor containing 12 C2H2-type zinc finger domains. Studies have shown that ZNF433 plays significant biological roles in various diseases and tumors. For example, ZNF433 is associated with multiple sclerosis (MS), potentially influencing leukotriene-mediated inflammatory responses by regulating 5-lipoxygenase, thereby contributing to the onset and progression of MS (16). Additionally, ZNF433 is significantly upregulated in prostate cancer, where it promotes cancer cell proliferation, migration, and tumorigenicity by activating the β-catenin/TCF signaling pathway, making it a potential therapeutic target for prostate cancer (17). In contrast, ZNF433 expression is significantly downregulated in clear cell renal carcinoma (ccRCC), and its low expression is closely associated with advanced tumor stages, higher grades, increased metastasis risk, and poorer survival prognosis. The downregulation of ZNF433 may be regulated by DNA methylation and is linked to mutations in key genes such as BAP1, KDM5C, and ALOX5, suggesting its potential role as a tumor suppressor (12).

Although previous studies have provided preliminary insights into the function of ZNF433 in individual cancers, systematic research on ZNF433 in the pan-cancer context is still lacking. This study aims to comprehensively utilize multiple public databases (such as TCGA, GTEx, HPA, TIMER2, etc.) to systematically analyze the expression patterns, promoter methylation, genetic variations, and its role in the tumor immune microenvironment across various cancers. We have evaluated the potential of ZNF433 as a diagnostic and prognostic biomarker in cancer and further explored its underlying biological functions and associated signaling pathways through bioinformatics analyses.

## 2 Materials and methods

### 2.1 Bioinformatic analysis of ZNF433 genomic features and expression patterns

To systematically investigate the genomic characteristics and expression profiles of ZNF433, we conducted a multi-platform bioinformatic analysis. First, the genomic localization of ZNF433 was determined using the UCSC Genome Browser (GRCh38/hg38 assembly; Dec. 2013 release) by mapping the chromosomal regions (18). Subsequently, mRNA and protein annotations were retrieved from the NCBI Gene database, specifically utilizing the “Gene Function” section and the “RefSeq” module for comprehensive molecular characterization.

The TIMER 2.0 platform (http://timer.cistrome.org/) (19) was used to analyze differential expression between tumor tissues (TCGA cohorts) and adjacent normal tissues through its “Gene_DE” module. Expression values were normalized as log2-transformed Transcripts Per Kilobase Million (TPM), and statistical significance (Wilcoxon test) was visualized via box plots. Gray/white color coding distinguished datasets with available normal tissue data.

SangerBox (http://SangerBox.com/tool) (20) facilitated cross-database integration, comparing TCGA tumor data with normal tissue profiles from GTEx (36). Expression values underwent log2(x+1) transformation, and 34 cancer types meeting the minimum sample threshold (n ≥ 3) were retained for analysis using violin plots.

Pathological stage correlation was assessed via GEPIA2 (http://GEPIA2.cancer-pku.cn) (21) using stage-stratified TCGA data. The “Pathological Stage Plot” module generated violin plots (log2[TPM + 1] transformed) to illustrate expression trends across stages I-IV.

Protein-level investigations were performed through the HPA (https://www.proteinatlas.org) (22). Subcellular localization data were acquired from the “Subcellular Atlas” (23). Single-cell resolution expression patterns in normal/tumor microenvironments were extracted from the “Tissue Atlas”, “Single Cell Type Atlas” (24), and “Cell Line Atlas” modules (TMM-normalized NX values). Immunohistochemical images comparing testicular, hepatic, and renal carcinomas with matched normal tissues were obtained from the “Pathology Atlas” (25).

### 2.2 Pan-cancer methylation profiling of ZNF433

In this study, a comprehensive pan-cancer analysis of ZNF433 promoter methylation levels was performed using the University of Alabama at Birmingham (UAB) Cancer Data Analysis Portal (UALCAN; http://ualcan.path.uab.edu/) (26). Promoter methylation patterns of ZNF433 were systematically compared across diverse cancer types. Additionally, correlations between ZNF433 promoter methylation levels and clinicopathological parameters, including tumor stage and lymph node metastasis status, were assessed through the UALCAN platform.

### 2.3 Genetic alteration analysis of ZNF433 across cancers

We logged into the cBioPortal website (https://www.cBioPortal.org/) and selected “TCGA PanCancer Atlas Studies” in the “Quick Select” section (27, 28). Next, we clicked on the “Gene Queries” button. After entering ZNF433, we were able to query its genetic variation characteristics. The “Cancer Types Summary” module was used to collect mutation features such as copy number alterations, mutation rates, and pan-cancer type changes. We further examined the mutation sites using the “Mutations” module in cBioPortal. Additionally, we utilized the “Comparison” module to compare data on OS, disease-specific survival (DSS), progression-free survival (PFS), and (DFS) for TCGA tumors with or without mutations in the ZNF433 gene.

### 2.4 Survival prognosis analysis based on ZNF433 expression

The survival prognosis analysis was conducted using a multi-platform approach. To evaluate ZNF433’s prognostic value across TCGA tumors, we initially employed the “Survival Map” module in GEPIA2 (21) to obtain data on OS and DFS. Expression-based stratification divided samples into high (top 50%) and low (bottom 50%) expression cohorts using median expression cutoffs. Corresponding survival curves with log-rank P values were generated through GEPIA2’s “Survival Analysis” module.

To enhance the prognostic assessment, complementary analyses were performed using SangerBox 3.0, where the “Gene Expression Prognostic Analysis” module was employed to retrieve comprehensive survival data, including OS, DSS, disease-free interval (DFI), and progression-free interval (PFI) across multiple cancer types. This platform integrated standardized pan-cancer data from the UCSC Xena Browser, encompassing sample types such as primary blood-derived cancer (peripheral blood) from TCGA-LAML, cutaneous melanoma metastases from TCGA-SKCM, primary tumor specimens, and primary/recurrent bone marrow-derived blood cancers.

Gene expression values were log2(x+1) transformed prior to analysis. To ensure data reliability, we implemented strict inclusion criteria, which involved excluding samples with follow-up durations of less than 30 days, removing cancer types with fewer than 10 eligible samples, integrating high-quality prognostic data from a seminal Cell study (29), and supplementing the follow-up data with information from the TARGET consortium via the UCSC Cancer Browser.

Prognostic significance was determined through Cox proportional hazards regression analysis using the survival R package (v3.2-7). The coxph function was implemented to evaluate associations between ZNF433 expression and survival outcomes, with statistical significance assessed by log-rank testing. This multi-modal approach enabled a systematic evaluation of ZNF433’s prognostic potential across diverse clinical endpoints and cancer types.

### 2.5 Correlation analysis between ZNF433 expression and genomic features (TMB/MSI)

We analyzed the Spearman correlation between ZNF433 expression levels and TMB or MSI across multiple TCGA tumor samples using the SangerBox platform. TMB has emerged as a crucial predictive biomarker for immunotherapy response, particularly demonstrating clinical utility in forecasting the outcomes of immune checkpoint inhibitor therapies. Elevated TMB levels are strongly associated with improved efficacy in immunotherapeutic treatments (30). Meanwhile, MSI represents a distinct molecular phenotype caused by defective MMR mechanisms in tumor cells. As a hallmark of MMR deficiency, MSI status serves both as a critical diagnostic marker and as a therapeutic predictor in clinical oncology (31).

### 2.6 Association of ZNF433 with MMR, DNMTs, and RNA modification genes

We performed a multi-platform analysis to systematically evaluate the associations between ZNF433 expression and three molecular systems: (1) MMR components (MLH1, MSH2, MSH6, PMS2, EPCAM), (2) DNA methyltransferases (DNMT1, DNMT3A, DNMT3B), and (3) RNA modification regulators across pan-cancer datasets. The investigation utilized the TIMER2.0 and GEPIA2.0 platforms for MMR/DNMT analysis, complemented by Sangerbox-based exploration of 44 RNA epigenetic regulators spanning three modification types: m1A (10 genes), m5C (13 genes), and m6A (21 genes). Notably examined modifiers included m1A-associated TRMT61 complexes (TRMT61A/B, TRMT6/10C), m6A readers (YTHDC1, YTHDF1-3), and demethylases (ALKBH1/3), along with m5C regulators such as NOP2. All correlations were computed using Spearman’s rank-order analysis, selected for its non-parametric robustness in handling complex biological datasets.

### 2.7 Tumor immune microenvironment characterization linked to ZNF433

To explore the relationship between ZNF433 expression and immunity, we systematically correlated ZNF433 with various immunological features in the pan-cancer dataset Tumor Microenvironment using Sangerbox. These features included: immunoregulatory gene analysis, immune checkpoint gene analysis, immune infiltration analysis (ImmuneScore), and immune cell analysis (Timer).

First, we examined the relationship between ZNF433 expression and immunoregulatory genes. By downloading the uniformly normalized pan-cancer dataset TCGA TARGET GTEx (PANCAN, N = 19,131, G = 60,499) from the UCSC database, we extracted the ZNF433 gene along with 150 marker genes representing five immune pathway categories: chemokines (41), receptors (18), MHC (major histocompatibility complex) (21), immunoinhibitors (24), and immunostimulators (46). We then extracted the expression data for the ZNF433 gene and the marker genes for these five immune pathways for each sample. Additional filtering was applied to include only samples from primary solid tumors and primary blood-derived cancers (bone marrow and peripheral blood), while excluding all normal samples. Expression values were log2(x + 1)-transformed, and the Spearman correlation between ZNF433 and the marker genes for the five immune pathways was calculated.

Next, we analyzed the relationship between ZNF433 expression and immune checkpoint genes. We extracted the expression data for ZNF433 and 60 marker genes from two types of immune checkpoint pathways—Inhibitory (24) and Stimulatory (36)—for each sample in the pan-cancer dataset. The Spearman correlation between ZNF433 and immune checkpoint genes was then calculated.

Subsequently, we investigated the relationship between ZNF433 expression and immune infiltration, as well as immune cell composition. We extracted the expression data for the ZNF433 gene for each sample, along with the gene expression profiles of each tumor in the pan-cancer dataset. These profiles were mapped to GeneSymbol, and an immune infiltration analysis was performed using the R package ESTIMATE (version 1.0.13). ESTIMATE was used to calculate immune scores for each patient based on gene expression. For immune cell analysis, we re-evaluated infiltration scores for B cells, CD4+ T cells, CD8+ T cells, neutrophils, macrophages, and dendritic cells (DCs) in each tumor using the Timer method from the R package IOBR (version 0.99.9). All correlation analyses were performed using Spearman’s rank correlation to ensure the robustness and applicability of the analysis.

### 2.8 Pan-cancer diagnostic potential evaluation of ZNF433

Diagnostic analysis of ZNF433 was performed using the Xiantao Academic Tool (https://www.xiantao.love/). Expression data for ZNF433 were obtained from the GTEx and TCGA databases. The analysis utilized TPM format RNA-seq data, which were log2(x + 1)-transformed. The diagnostic potential of ZNF433 was evaluated using the pROC package (version 1.18.5).The ROC curve was plotted using the “ggplot2” package. The Area Under the Curve (AUC) was used to determine diagnostic accuracy: an AUC value between 0.5 and 0.7 indicates low accuracy, between 0.7 and 0.9 suggests moderate accuracy, and above 0.9 represents high accuracy.

### 2.9 Drug sensitivity analysis associated with ZNF433 status

The compound activity data and RNA-seq expression profiles from CellMiner™ for the NCI-60 were downloaded to analyze the drug sensitivity of ZNF433 in pan-cancer (https://discover.nci.nih.gov/cellminer/home.do) (32, 33). FDA-approved drugs or those under clinical trials were selected for analysis. A custom R script was implemented in R version 4.3.2 using key packages, including limma (v3.52.4) for probe-level expression processing, impute (v1.76.0) for missing value imputation, and ggplot2 (v3.5.1) and ggpubr (v0.6.0) for visualization.

### 2.10 Functional enrichment analysis of ZNF433-related pathways

The STRING database (https://string-db.org/, version 11.0) was utilized to predict protein-protein interactions (PPIs) for human ZNF433 (34–36). We searched for ZNF433 by entering its gene name and applied the following settings: (1) Network type: All networks; (2) Edge evidence: Meaningful evidence; (3) Active interaction sources: Experimental; (4) Minimum required interaction score: Low confidence (0.150); and (5) Display up to 50 interactors in the first shell, showing the three interactors with the highest custom value in the second shell. This approach resulted in a PPI network consisting of 56 experimentally validated ZNF433-interacting proteins.

Subsequently, we used GEPIA2 to identify the top 100 genes related to ZNF433 in both TCGA tumor and normal tissues, selecting the five genes most highly correlated with ZNF433. We then employed TIMER2 to generate a heatmap showing the expression correlation between ZNF433 and these selected genes across various cancer types.

Finally, to explore the functional roles and metabolic pathways associated with the ZNF433-binding genes, we conducted Gene Ontology (GO) (37, 38) and Kyoto Encyclopedia of Genes and Genomes (KEGG) (39, 40) pathway analyses using Sangerbox.

### 2.11 Experimental validation

#### 2.11.1 Cell line

Human renal proximal tubule epithelial cells (HK-2) and renal cell carcinoma cell lines (A-498, 786-O, and Caki-1) were used in this study. The cells were cultured in high-glucose DMEM medium supplemented with 10% fetal bovine serum and maintained in a 5% CO_2_ incubator.

#### 2.11.2 Sample collection

This study included 25 patients who underwent surgical treatment (including robot-assisted laparoscopic/laparoscopic partial nephrectomy or radical nephrectomy) at the First Affiliated Hospital of Gannan Medical University between September 1, 2020, and January 31, 2025, and were pathologically diagnosed with clear cell renal carcinoma. The specimens obtained during surgery were stored in cryopreservation tubes according to standard procedures and kept in a -80°C freezer at the research institute. The study protocol was approved by the Ethics Committee of the First Affiliated Hospital of Gannan Medical University (Ethics No. 22SC-2024-355), and informed consent was obtained from the parents or guardians of all participating patients. This study was conducted in accordance with the Declaration of Helsinki.

#### 2.11.3 Quantitative PCR

Total RNA was extracted from samples using the TransZol Up Plus RNA Kit (TransGen Biotech Co., Ltd., Beijing, China; https://www.transgen.com.cn/) according to the manufacturer’s instructions. cDNA was synthesized using the TransScript^®^ One-Step gDNA Removal and cDNA Synthesis SuperMix (TransGen Biotech Co., Ltd.). qPCR was conducted with the PerfectStart^®^ Green qPCR SuperMix (TransGen Biotech Co., Ltd.) to assess the mRNA expression levels of ZNF433 in both cancerous and paired non-cancerous tissues. The forward primer for ZNF433 was 5’-gaacatatatgtagagtacg-3’, and the reverse primer was 5’-cctacttctccatacacact-3’.

#### 2.11.4 Western blot analysis

To assess the expression level of ZNF433 protein, samples were first lysed using RIPA buffer, and protein concentrations were determined using the BCA protein assay kit. Following protein quantification, equal amounts of protein were loaded onto an SDS-PAGE gel for separation, after which the proteins were transferred to a PVDF membrane. The membrane was subsequently blocked with 5% non-fat milk solution. The primary antibody against ZNF433 (anti-ZNF433, orb1439228, Biorbyt, Cambridge, United Kingdom), diluted 1:1000, was applied to the membrane and incubated overnight at 4°C. Afterward, the membrane was incubated with a secondary antibody at room temperature for 1 hour, and the expression of the target protein was detected using an enhanced chemiluminescence detection kit.

#### 2.11.5 Immunohistochemistry

First, tissue slides were baked at 65°C for 2 hours. Deparaffinization was performed using xylene, followed by dehydration through a gradient series of ethanol. To inactivate endogenous peroxidase, the slides were incubated in 0.3% hydrogen peroxide in methanol solution at room temperature for 15 minutes. Antigen retrieval was then performed using 0.01 mol/L citrate buffer (pH 6.0). Afterward, the slides were incubated with 10% normal goat serum at 37°C for 30 minutes to block non-specific binding. Following serum removal, the slides were incubated with diluted primary antibody (anti-ZNF433, orb536701, Biorbyt, Cambridge, United Kingdom) at 37°C for 2 hours. After washing, a biotin-labeled secondary antibody was applied, followed by incubation at 37°C for 30 minutes. The slides were then developed with DAB chromogenic solution, with the reaction being monitored until optimal staining was achieved, after which the reaction was terminated with PBS. The nuclei were counterstained with hematoxylin for 3 minutes and rinsed. Images were analyzed using ImageJ software.

### 2.12 Statistical analysis

Bioinformatics statistical analysis was performed using R version 4.3.2, with a significance level set at P < 0.05 to determine statistical significance.

## 3 Results

### 3.1 Expression profile of ZNF433 across pan-cancer

**Figure 1:**
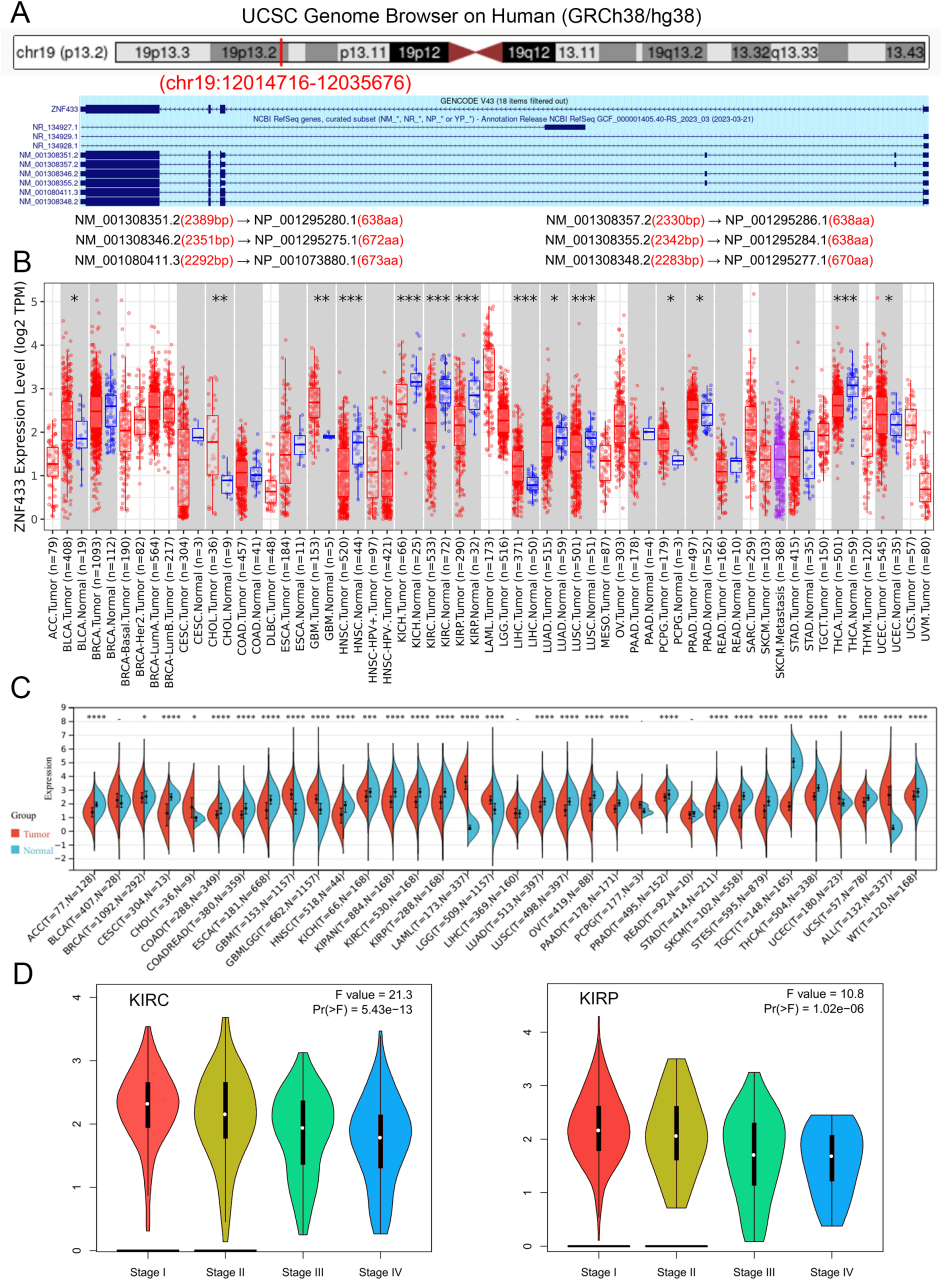
Characteristics of ZNF433 and its expression levels in normal tissues and cancers. **(A)** Genomic localization of the human ZNF433 gene. **(B)** mRNA expression levels of ZNF433 in various tumor types from the TCGA database, analyzed using TIMER2. **(C)** Differential mRNA expression levels of ZNF433 in multiple tumors and normal tissues, assessed using data from the TCGA and GTEx databases via SangerBox. **(D)** Expression levels of the ZNF433 gene across different pathological stages of KIRC and KIRP in the TCGA database. (*P < 0.05, **P < 0.01, ***P < 0.001, ****P < 0.0001).

The analytical process is presented in **Supplementary Figure 1**. We aim to investigate the role of the ZNF433 gene in human cancer. This gene exhibits six mRNA transcript variants, namely NM_001308351.2, NM_001308357.2, NM_001308346.2, NM_001308355.2, NM_001080411.3, and NM_001308348.2. Additionally, it encodes six zinc finger protein 433 isoforms: NP_001295280.1, NP_001295286.1, NP_001295275.1, NP_001295284.1, NP_001073880.1, and NP_001295277.1 (**Figure 1A**).

To further investigate the expression patterns of the ZNF433 gene in human cancers, we conducted expression analyses using the TIMER2.0 online database. **Figure 1B** presents the differential expression of ZNF433 across various cancer types from the TCGA database. Compared to adjacent normal tissues, significant differences in ZNF433 expression were observed in BLCA, CHOL, GBM, HNSC, KICH, KIRC, KIRP, LIHC, LUAD, LUSC, PCPG, PRAD, THCA, and UCEC.

Due to the limited availability of normal specimens in the TCGA tumor samples, we utilized SangerBox to integrate transcriptomic data from three datasets (HPA, GTEx, and FANTOM5) to analyze ZNF433 expression in 34 cancer types. The results revealed significant upregulation of ZNF433 expression in seven tumor types, including GBM, GBMLGG, LGG, UCEC, ALL, LAML, and CHOL. Conversely, significant downregulation of ZNF433 expression was observed in 23 tumor types, including BRCA, CESC, LUAD, ESCA, STES, KIRP, KIPAN, COAD, COADREAD, PRAD, STAD, HNSC, KIRC, LUSC, WT, SKCM, THCA, OV, PAAD, TGCT, UCS, ACC, and KICH (**Figure 1C**). Detailed data can be found in **Supplementary Table 1**.

Furthermore, utilizing the HPA database, we investigated the cellular localization of ZNF433 and its expression levels across various tissues, single-cell types, and cell lines. As shown in **Supplementary Figure 2A**, ZNF433 was localized to the nucleolus within cells. Immunofluorescence staining of the human RT-4 cell line further confirmed its nuclear localization (**Supplementary Figure 2B**).

Next, we analyzed the expression patterns of ZNF433 in multiple normal tissues. Although ZNF433 expression was generally low in most normal tissues, it remained detectable (NX > 1), indicating relatively low tissue specificity of ZNF433 mRNA (**Supplementary Figure 2C**). Additionally, an assessment of its expression across different single-cell types (**Supplementary Figure 2D**) revealed a significant increase in ZNF433 expression in early and late spermatids, while other cell types—including oligodendrocytes, skeletal muscle cells, proximal intestinal cells, monocytes, and dendritic cells—showed no detectable expression.

Finally, we examined ZNF433 expression in various cell lines. No significant increase was observed in either tumor or non-tumor cell lines (**Supplementary Figure 2E**). These findings highlight the differential expression patterns of ZNF433 mRNA in normal tissues, as well as in tumor and non-tumor cell lines, offering a comprehensive overview of its expression specificity.

**Figure 2:**
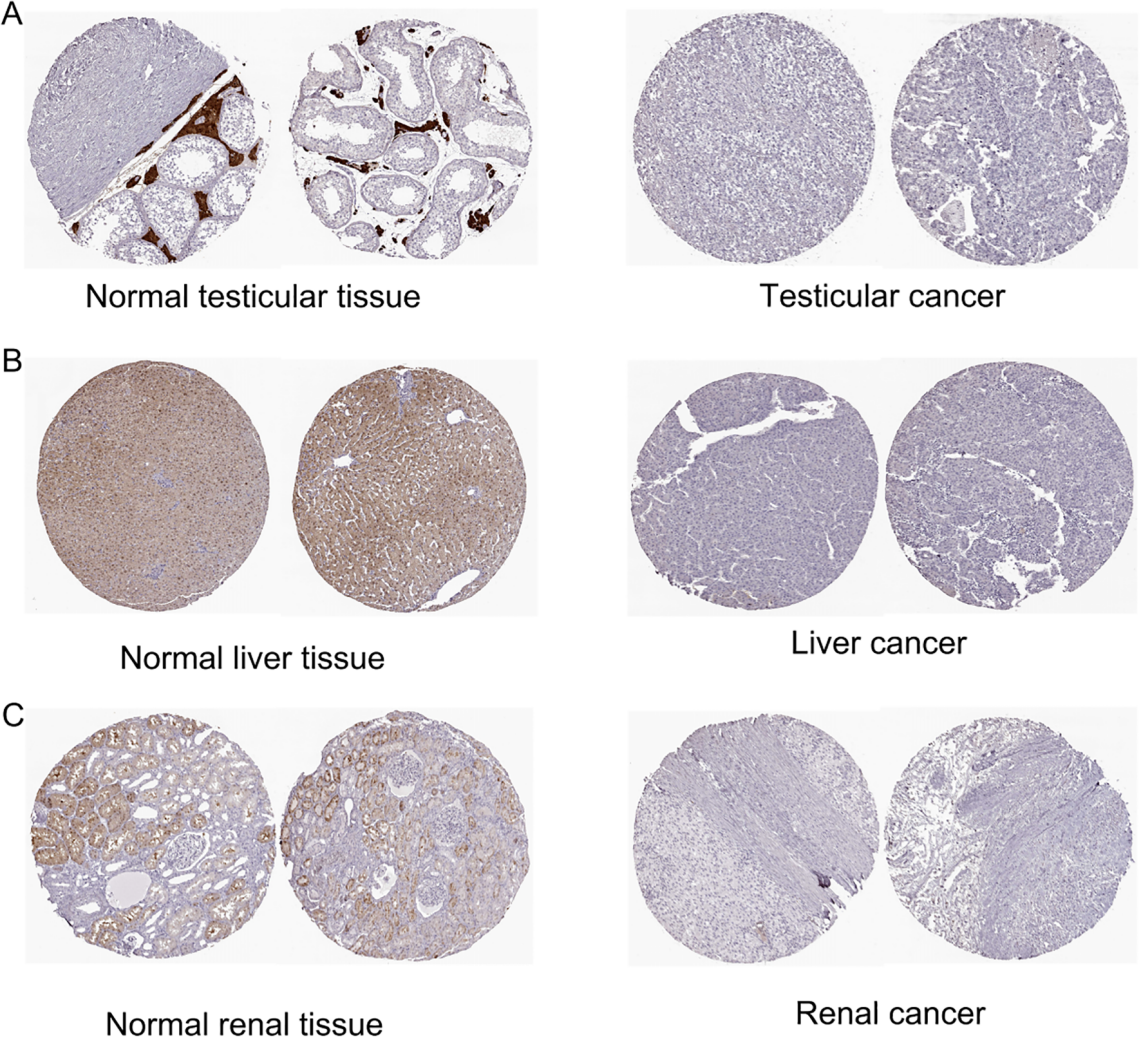
Representative immunohistochemical staining of ZNF433 in HPA. **(A)** Normal testicular tissue and testicular cancer. **(B)** Normal liver tissue and liver cancer. **(C)** Normal kidney tissue and kidney cancer.

Based on our investigation of ZNF433 protein expression in the HPA database, we provide immunohistochemistry (IHC) results of ZNF433 in tumor and normal tissues. According to the analysis results, it is shown that in normal testicular tissue, ZNF433 exhibits high staining intensity, while in normal liver and kidney tissue, its staining level is moderate. However, in the corresponding tumor tissue, ZNF433 expression demonstrates negative or weak staining characteristics, which indicate that the expression of ZNF433 protein is downregulated in these types of cancers (**Figure 2**).

In addition, the relationship between ZNF433 expression levels and various cancer pathological stages was analyzed using GEPIA2. The expression of ZNF433 was significantly associated with the pathological stages of KIRC and KIRP (**Figure 1D**). Overall, these findings suggest that ZNF433 may play a crucial role in different cancers.

### 3.2 Pan-cancer methylation landscape of ZNF433

**Figure 3:**
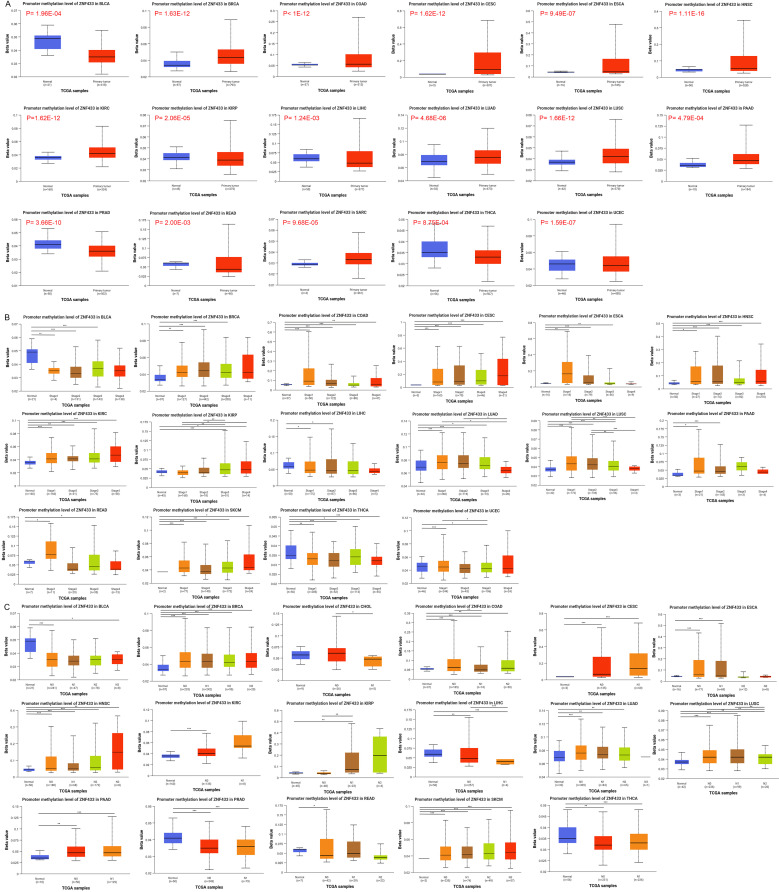
Promoter methylation levels of ZNF433 in cancer. **(A)** Comparison of ZNF433 promoter methylation levels between tumor and normal samples. **(B)** ZNF433 promoter methylation levels across different tumor stages compared to normal samples. **(C)** ZNF433 promoter methylation levels in tumors with varying lymph node metastasis statuses compared to normal samples.*p < 0.05, **p < 0.01, ***p < 0.001.

Through the UALCAN portal, we assessed the methylation levels of the ZNF433 promoter in tumor and normal tissues (26). We found that, compared to normal tissues, the promoter methylation levels of ZNF433 were significantly elevated in BRCA, COAD, CESC, ESCA, HNSC, KIRC, KIRP, LIHC, LUAD, LUSC, PAAD, READ, SARC, and UCEC, while they were lower in BLCA, PRAD, and THCA (**Figure 3A**). Additionally, increased promoter methylation levels of ZNF433 were observed in tumor tissues across various stages of BRCA, COAD, CESC, HNSC, KIRC, KIRP, and LUAD (**Figure 3B**). Furthermore, higher methylation levels were noted in tumor tissues with lymph node metastasis in BRCA, COAD, CESC, HNSC, LUAD, LUSC, PAAD, and SKCM compared to normal tissues (**Figure 3C**). These findings suggest that the methylation of the ZNF433 promoter is negatively correlated with its mRNA expression, indicating that ZNF433 may act as a tumor suppressor gene in cancer.

Using the UALCAN portal, we evaluated the methylation levels of the ZNF433 promoter in both tumor and normal tissues. Compared to normal tissues, ZNF433 promoter methylation was significantly elevated in BRCA, COAD, CESC, ESCA, HNSC, KIRC, KIRP, LIHC, LUAD, LUSC, PAAD, READ, SARC, and UCEC, while lower levels were observed in BLCA, PRAD, and THCA (**Figure 3A**). Increased promoter methylation levels of ZNF433 were also detected in tumor tissues at various stages of BRCA, COAD, CESC, HNSC, KIRC, KIRP, and LUAD (**Figure 3B**). Moreover, higher methylation levels were associated with lymph node metastasis in tumors of BRCA, COAD, CESC, HNSC, LUAD, LUSC, PAAD, and SKCM compared to normal tissues (**Figure 3C**). These findings suggest a negative correlation between ZNF433 promoter methylation and its mRNA expression, supporting the hypothesis that ZNF433 may function as a tumor suppressor gene in cancer.

### 3.3 Genetic alteration patterns of ZNF433 in human cancers

**Figure 4:**
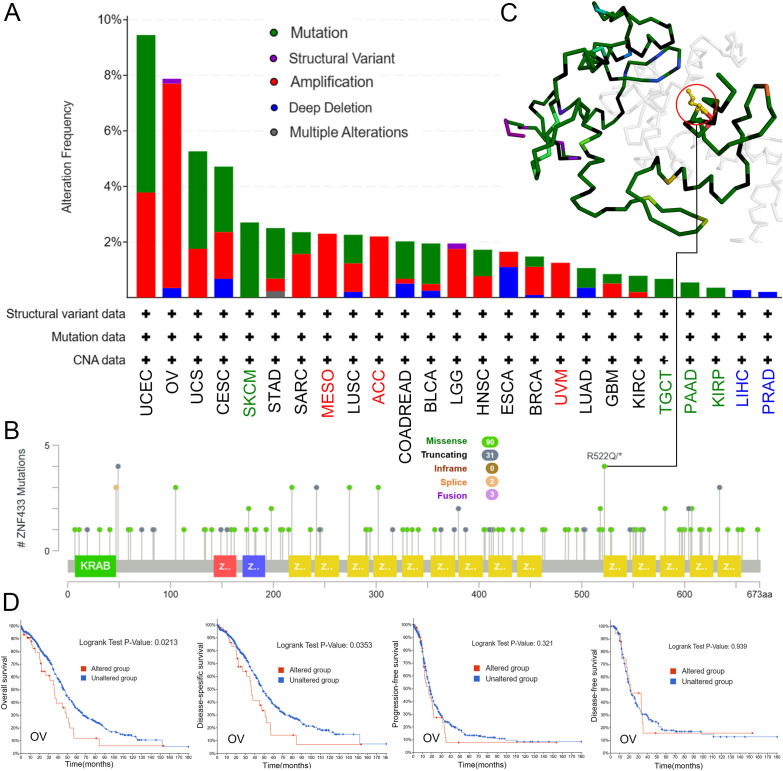
Analysis of ZNF433 gene alterations across different tumor types in TCGA. **(A)** Frequency of ZNF433 alterations, categorized by mutation type. **(B)** Distribution of mutation sites in the ZNF433 gene. **(C)** The location of the R522Q mutation within the 3D structure of ZNF433. **(D)** Examination of the potential correlations between OS, DSS, PFS, DFS, and mutation status in OV using the cBioPortal tool.

We assessed the genetic alteration status of the ZNF433 gene using the cBioPortal database, examining mutations, structural variations, amplifications, deep deletions, and multiple alterations. Our analysis revealed that in UCEC patients, ZNF433 exhibited the highest alteration frequency, at 9.45% (50 cases), with mutations being the predominant alteration, accounting for 5.67% (30 cases). Additionally, mutations were the only alteration type observed in all cases of SKCM, TGCT, PAAD, and KIRP, while amplifications were exclusively found in MESO, ACC, and UVM cases, and deep deletions were the sole alteration in all LIHC and PRAD cases (**Figure 4A**). The distribution of ZNF433 mutations is shown in **Figure 4B**. The most frequent mutation was a missense mutation, particularly the R522Q/* mutation, present in 4 out of 126 cases. This mutation results in the substitution of aspartic acid with glutamine at position 522. The R522Q/* site is also highlighted in the three-dimensional structure of the ZNF433 protein (**Figure 4C**). Furthermore, we explored the potential association between ZNF433 alterations and clinical survival rates across different cancer types. As shown in **Figure 4D**, OV cases with ZNF433 alterations had a worse prognosis in terms of (OS; P = 0.0213) and (DSS; P = 0.0353) compared to those without alterations, though no significant correlation was found for progression-free survival (PFS; P = 0.321) or (DFS; P = 0.939). Based on these findings, we hypothesize that alterations in ZNF433 may contribute to cancer progression.

### 3.4 Prognostic value of ZNF433 expression across cancer types

**Figure 5:**
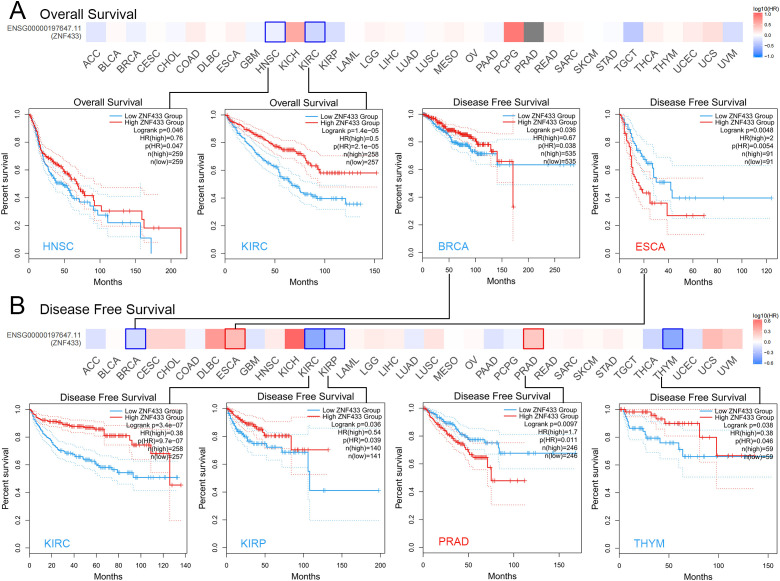
Correlation analysis between ZNF433 expression and prognosis in cancers from the TCGA database using GEPIA2. **(A, B)** The survival maps showing significant results are presented.

Using the median expression level as a cutoff, tumor samples were divided into high-expression and low-expression groups to examine the correlation between gene expression and prognosis. Survival analysis revealed that high ZNF433 expression was associated with improved OS in HNSC and KIRC (P < 0.05) (**Figure 5A**). Additionally, as shown in **Figure 5B**, high ZNF433 expression in ESCA and PRAD was linked to poorer DFS (P < 0.05), while high expression in BRCA, KIRC, KIRP, and THYM was associated with better DFS (P < 0.05).

To further investigate the potential of ZNF433 as a prognostic biomarker in cancer, we used SangerBox to analyze survival data from pan-cancer datasets, including TCGA, TARGET, and GTEx. This analysis covered OS, DSS, DFI, and PFI, with the results presented as forest plots. A hazard ratio (HR) > 1 indicates a risk factor for survival, while an HR < 1 suggests a protective effect on survival.

The OS analysis revealed that high ZNF433 expression was associated with poor prognosis in six tumor types: TCGA-GBMLGG (N = 619, p = 7.9e-10, HR = 2.36 [1.80, 3.11]), TCGA-LGG (N = 474, p = 0.04, HR = 1.63 [1.02, 2.60]), TARGET-LAML (N = 142, p = 3.0e-5, HR = 1.45 [1.21, 1.72]), TCGA-PRAD (N = 492, p = 0.02, HR = 10.51 [1.47, 75.33]), TCGA-SKCM-P (N = 97, p = 0.05, HR = 2.01 [1.01, 4.02]), and TCGA-PCPG (N = 170, p = 0.01, HR = 13.39 [1.81, 99.09]). In contrast, low expression of ZNF433 was linked to poor prognosis in four tumor types: TCGA-KIRP (N = 276, p = 3.6e-5, HR = 0.43 [0.29, 0.64]), TCGA-KIPAN (N = 855, p = 4.2e-12, HR = 0.53 [0.45, 0.64]), TCGA-KIRC (N = 515, p = 2.0e-7, HR = 0.58 [0.47, 0.72]), and TCGA-UVM (N = 74, p = 0.02, HR = 0.36 [0.15, 0.88]) (**Supplementary Figure 3A**).

The DSS analysis indicated that high expression of ZNF433 was associated with poor prognosis in three tumor types: TCGA-GBMLGG (N = 598, p = 5.3e-9, HR = 2.52 [1.85, 3.43]), TCGA-LGG (N = 466, p = 0.02, HR = 1.77 [1.09, 2.87]), and TCGA-PCPG (N = 170, p = 0.01, HR = 26.85 [2.15, 335.88]). Conversely, low expression was associated with poor prognosis in five tumor types: TCGA-KIRP (N = 272, p = 8.3e-10, HR = 0.24 [0.15, 0.38]), TCGA-KIPAN (N = 840, p = 1.4e-20, HR = 0.37 [0.30, 0.46]), TCGA-KIRC (N = 504, p = 1.6e-12, HR = 0.41 [0.32, 0.53]), TCGA-THYM (N = 117, p = 0.02, HR = 0.09 [0.01, 0.72]), and TCGA-UVM (N = 74, p = 0.02, HR = 0.32 [0.12, 0.85]) (**Supplementary Figure 3B**).

The DFI analysis showed that high expression of ZNF433 was associated with poor prognosis in TCGA-CESC (N = 171, p = 7.6e-3, HR = 1.74 [1.14, 2.64]), while low expression correlated with poor prognosis in TCGA-BRCA (N = 904, p = 0.02, HR = 0.66 [0.47, 0.93]) and TCGA-KIPAN (N = 319, p = 0.02, HR = 0.59 [0.39, 0.91]) (**Supplementary Figure 3C**).

The PFI analysis revealed that high ZNF433 expression was associated with poor prognosis in five tumor types: TCGA-GBMLGG (N = 616, p = 4.3e-8, HR = 1.98 [1.55, 2.52]), TCGA-LGG (N = 472, p = 0.02, HR = 1.55 [1.07, 2.26]), TCGA-ESCA (N = 173, p = 0.04, HR = 1.34 [1.01, 1.76]), TCGA-PRAD (N = 492, p = 0.02, HR = 1.93 [1.12, 3.32]), and TCGA-LIHC (N = 340, p = 0.04, HR = 1.33 [1.01, 1.76]). Low expression was linked to poor prognosis in four tumor types: TCGA-KIRP (N = 273, p = 6.8e-6, HR = 0.43 [0.29, 0.62]), TCGA-KIPAN (N = 845, p = 4.2e-18, HR = 0.46 [0.38, 0.55]), TCGA-KIRC (N = 508, p = 1.2e-13, HR = 0.46 [0.37, 0.57]), and TCGA-THYM (N = 117, p = 0.03, HR = 0.51 [0.28, 0.93]) (**Supplementary Figure 3D**).

These results suggest that ZNF433 expression is closely associated with prognosis across various cancers and may serve as a valuable prognostic marker.

### 3.5 ZNF433 expression associates with TMB and MSI

**Figure 6:**
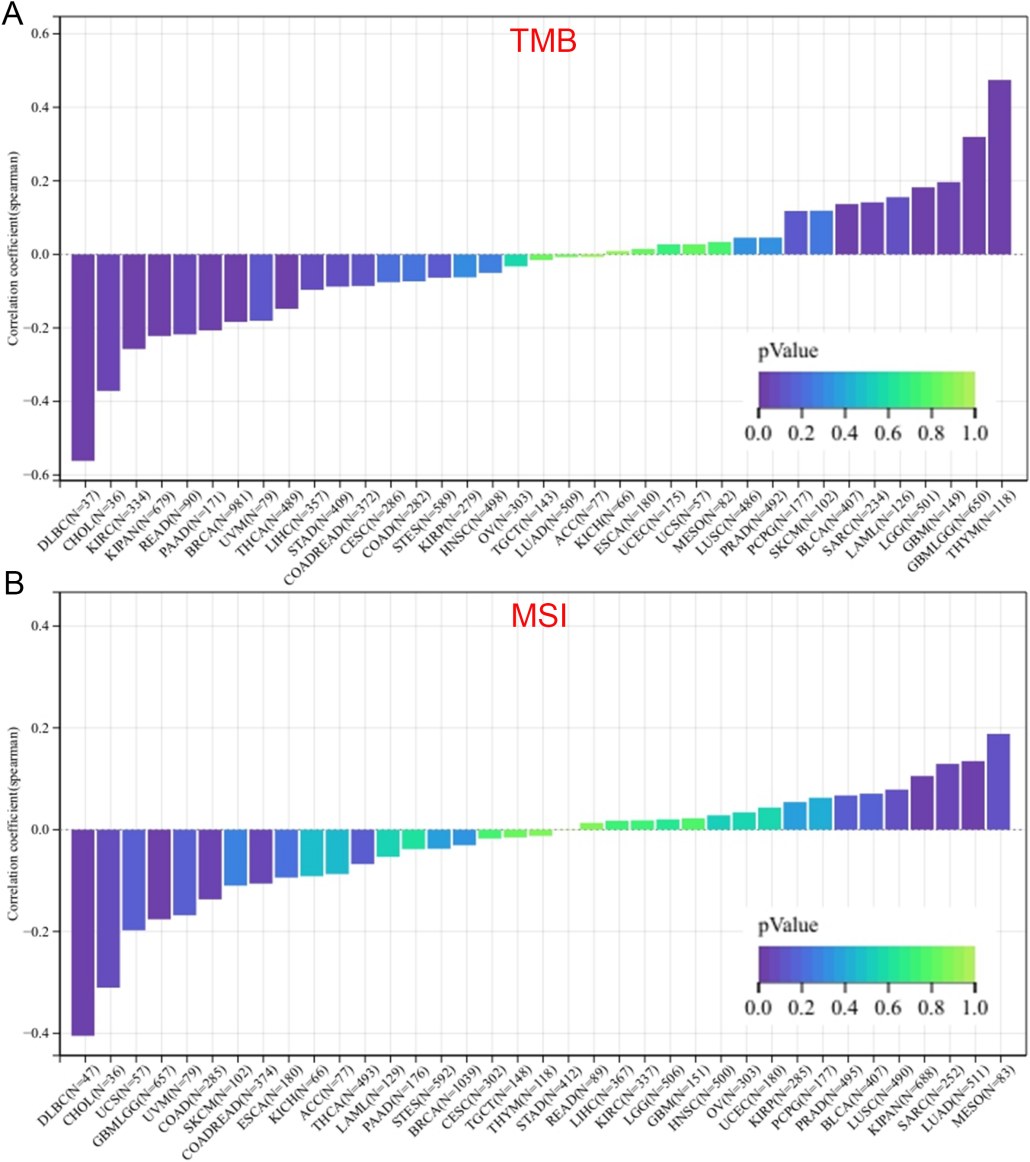
Correlation between ZNF433 expression and TMB **(A)** and MSI **(B)** across pan-cancer.

Numerous studies have demonstrated that TMB and MSI are important indicators for evaluating the response to cancer immunotherapy and prognosis (41–43). Therefore, we assessed the correlation between ZNF433 gene expression and TMB/MSI across pan-cancer. As shown in **Figure 6**, we observed a significant positive correlation between ZNF433 expression and TMB in GBM (P < 0.05), GBMLGG (P < 0.001), LGG (P < 0.001), SARC (P < 0.05), THYM (P < 0.001), and BLCA (P < 0.01). Conversely, ZNF433 expression showed a significant negative correlation with TMB in BRCA (P < 0.001), KIPAN (P < 0.001), KIRC (P < 0.001), THCA (P < 0.01), READ (P < 0.05), PAAD (P < 0.01), CHOL (P < 0.05), and DLBC (P < 0.001) across eight tumor types. Additionally, ZNF433 expression demonstrated a significant positive correlation with MSI in LUAD (P < 0.01), SARC (P < 0.05), and KIPAN (P < 0.01), while showing a significant negative correlation in GBMLGG (P < 0.001), COAD (P < 0.05), COADREAD (P < 0.05), and DLBC (P < 0.01). For detailed data on TMB and MSI, refer to **Supplementary Table 2**.

### 3.6 Coordinated dysregulation of ZNF433 with MMR, DNMTs, and RNA modification machinery

**Figure 7:**
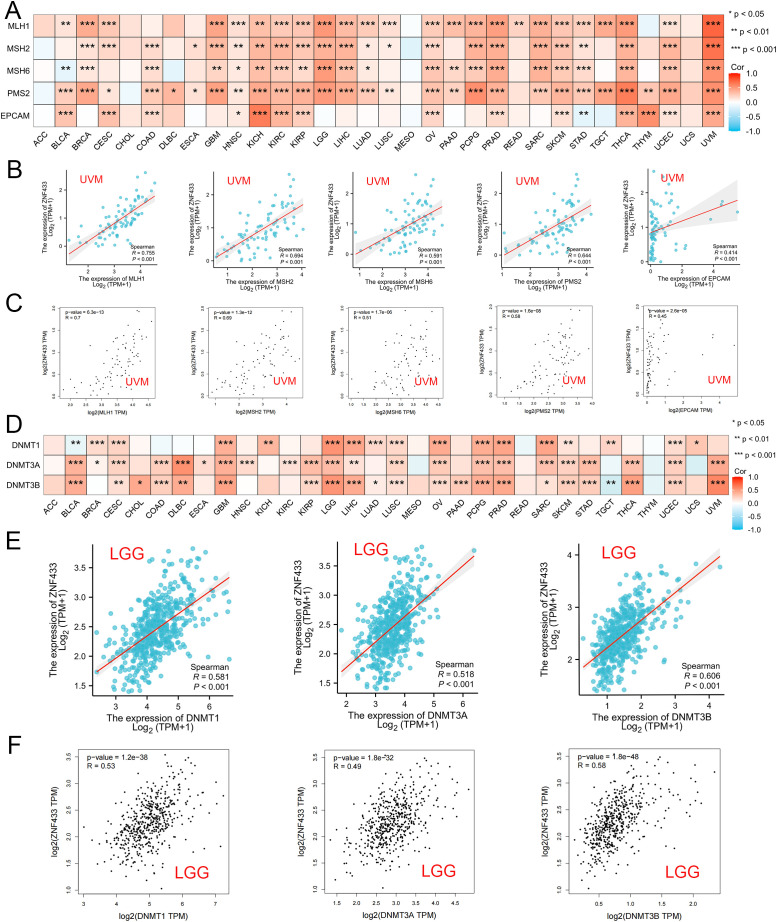
Correlation between ZNF433 expression and MMR and DNMT gene expression across various cancers. **(A)** The heatmap shows the Spearman correlation between ZNF433 expression and the levels of five MMR genes (MLH1, MSH2, MSH6, PMS2, and EPCAM) in pan-cancer. The correlation between ZNF433 expression and MMR gene expression in UVM is shown in TIMER1.2 **(B)** and confirmed through GEPIA2 **(C)**. **(D)** The heatmap illustrates the Spearman correlation between ZNF433 expression and the expression of three DNA methyltransferase genes (DNMT1, DNMT3A, and DNMT3B) in pan-cancer. The correlation between ZNF433 expression and DNA methyltransferase gene expression in LGG is shown in TIMER2 **(E)** and confirmed through GEPIA2 **(F)**.

Using the TIMER2.0 and GEPIA2.0 databases, we evaluated the relationship between ZNF433 and MMR genes, as well as DNA methyltransferase gene expression levels. The role of ZNF433 in tumor development was further verified through the GEPIA2.0 database. The results showed that in human cancers, ZNF433 was positively correlated with five MMR genes, including MLH1, MSH2, MSH6, PMS2, and EPCAM (**Figure 7A**), particularly in UVM (**Figures 7B, C**). Additionally, ZNF433 expression was closely associated with the levels of DNMT1, DNMT3A, and DNMT3B in various cancer types, including BRCA, CESC, GBM, LGG, LIHC, LUSC, OV, PCPG, PRAD, SARC, SKCM, and UCEC (**Figures 7D–F**).

**Figure 8:**
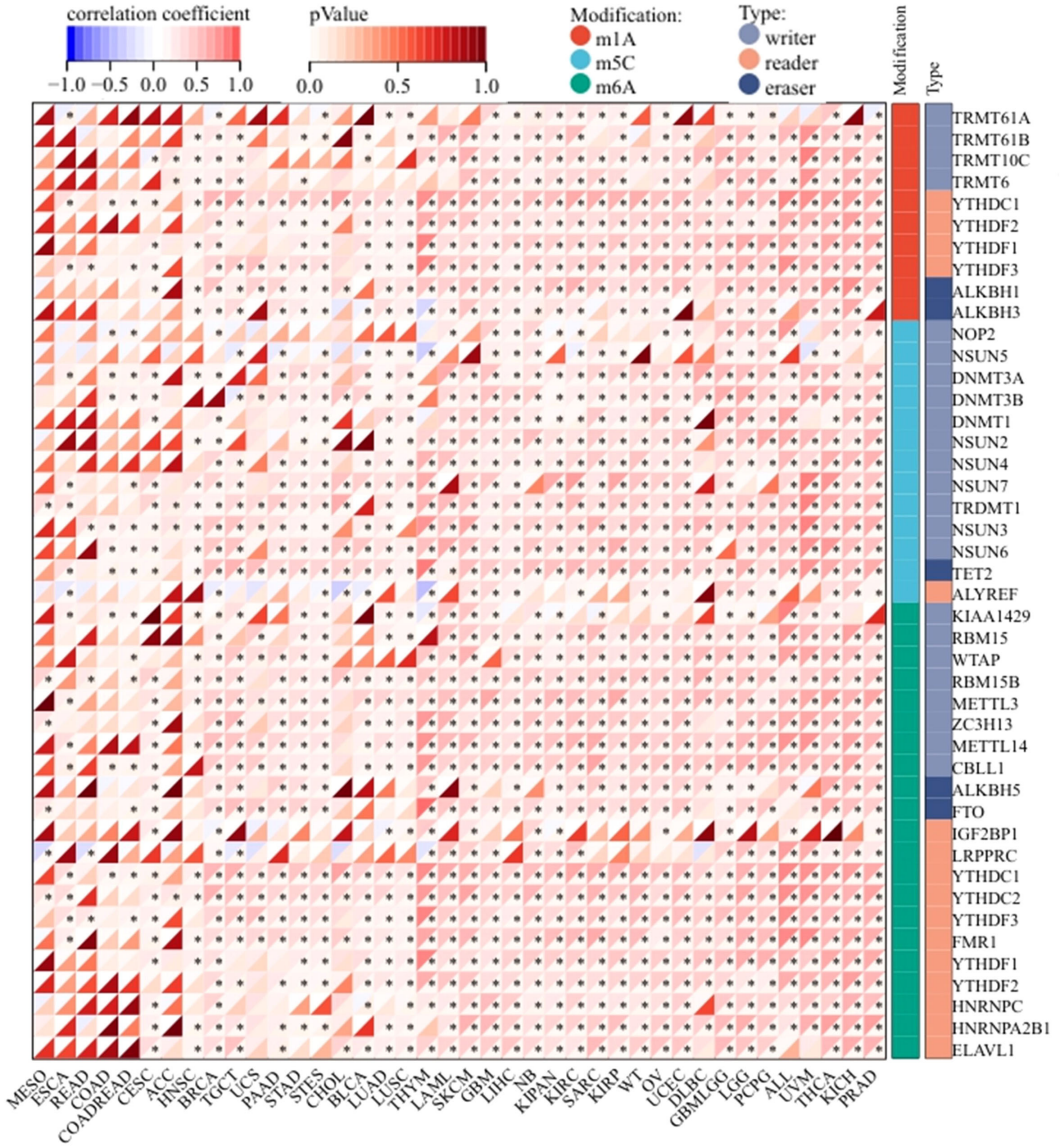
Correlation between ZNF433 gene expression and the levels of common RNA modification genes across pan-cancer.

We next evaluated the correlation between ZNF433 expression and 44 common RNA modification regulators across different cancer types. Notably, ZNF433 expression showed positive correlations with most RNA modification regulators, such as YTHDF3, DNMT3B, TET2, METTL14, and YTHDC1, in BRCA, THYM, LAML, SKCM, KIPAN, KIRC, ALL, UVM, THCA, KICH, and PRAD (**Figure 8**). These findings suggest that ZNF433 may influence tumor development by regulating DNA damage, DNA methylation, or RNA modification.

### 3.7 ZNF433 modulates tumor immune microenvironment composition

To investigate the relationship between ZNF433 expression and immunity, we conducted a systematic analysis correlating ZNF433 with immune features in the tumor microenvironment, commonly used in cancer research datasets (44–46). These features include immune regulatory gene analysis, immune checkpoint gene analysis, immune infiltration analysis, and immune cell analysis. Our goal is to comprehensively understand the role of ZNF433 in immune regulation and uncover its potential mechanisms in immune therapy.

Immunoregulatory gene analysis involves studying genes associated with immune system function and regulation. It explores gene variations, expression levels, and genetic polymorphisms linked to immune responses, aiming to better understand their roles and regulatory mechanisms within the immune system. This analysis can provide deeper insights into how the immune system impacts diseases and their treatment (47–49).

Immune checkpoint genes are critical regulatory molecules in the immune system that help modulate immune responses. These include IL4, CTLA-4, CD80, and others, which maintain immune balance and self-tolerance under normal conditions. However, in disease states like cancer, tumor cells can manipulate immune checkpoint pathways to evade immune attacks. Immune checkpoint inhibitors, which block these pathways, have become a widely used therapeutic strategy in cancer treatment (50–52).

In summary, immunoregulatory gene analysis focuses on genes related to immune function and regulation, while immune checkpoint genes are pivotal signaling molecules closely linked to immune tolerance and immune responses.

Through our analysis of the relationship between ZNF433 expression and both immunoregulatory and immune checkpoint genes (**Supplementary Figures 4**, **5**), we observed a significant negative correlation between ZNF433 and most of these genes in various cancer types, including BLCA, GBM, KIPAN, KIRC, SARC, and THCA. These findings suggest that ZNF433 may suppress the expression of immune modulators in these cancers, implying its potential involvement in anti-tumor immune responses or immune escape mechanisms.

However, in cancer types such as DLBC, PAAD, and PRAD, ZNF433 exhibited a significant positive correlation with most immunoregulatory genes and immune checkpoint genes, suggesting a regulatory role for ZNF433 in the development of these cancers. Specifically, ZNF433 may influence molecules or pathways in the immune system, thereby impacting the growth and spread of cancer cells. Further research is required to validate and expand our understanding of the mechanisms underlying this association.

Immune infiltration analysis is a quantitative analysis of the immune cell infiltration in tissue or tumor samples to evaluate the degree of immune response. It can reveal the distribution of immune cells in the tumor microenvironment or infection site, as well as their impact on disease progression and prognosis. Immunoscoring is a method of assessing the immune cell infiltration status and the degree of immune response. The results of this scoring can be used to evaluate the extent of immune cell infiltration in the tumor microenvironment and understand the impact of immune response on tumor development and treatment response. A higher immune score typically indicates a higher degree of immune cell infiltration and is associated with better prognosis and response to immune therapy (53–55). On the other hand, immune cell analysis focuses on the function, phenotype, and regulatory mechanisms of specific types of immune cells in the immune system. It investigates the development, activation, effector functions, and regulatory mechanisms of immune cells, revealing their roles in immune responses and the occurrence and development of disease. Immune cell analysis and immune infiltration analysis are interconnected, collectively helping us understand the role of the immune system in both healthy and diseased states (56).

In our immune infiltration analysis, we scored immune infiltration in 10,180 tumor samples across 44 cancer types. As shown in **Supplementary Figure 6**, we observed a significant correlation between ZNF433 expression and immune infiltration in 27 cancer types. Specifically, ZNF433 expression was positively correlated with immune infiltration in TCGA-DLBC (N = 46, R = 0.32, P = 0.03), while a significant negative correlation was found in 26 other cancer types, including TCGA-GBM (N = 152, R = -0.51, P = 2.0e-11).

In our immune cell analysis, we performed immune infiltration scoring across 44 tumor types, covering a total of 10,180 tumor samples. As shown in **Supplementary Figure 7**, we found a significant correlation between gene expression and immune infiltration in 32 cancer types, including TCGA-BLCA, TCGA-BRCA, TCGA-COADREAD, TCGA-DLBC, TCGA-ESCA, TCGA-GBM, TCGA-GBMLGG, TCGA-HNSC, TCGA-KICH, TCGA-KIPAN, TCGA-KIRC, TCGA-KIRP, TCGA-LGG, TCGA-LIHC, TCGA-LUAD, TCGA-LUSC, TCGA-MESO, TCGA-OV, TCGA-PAAD, TCGA-PCPG, TCGA-PRAD, TCGA-READ, TCGA-SARC, TCGA-SKCM-M, TCGA-SKCM-P, TCGA-SKCM, TCGA-STAD, TCGA-STES, TCGA-THCA, TCGA-THYM, TCGA-UCS, and TCGA-UVM. We further visualized the most statistically significant results using the scatter plot in **Supplementary Figure 8**.

### 3.8 Diagnostic potential of ZNF433 in pan-cancer early detection

**Figure 9:**
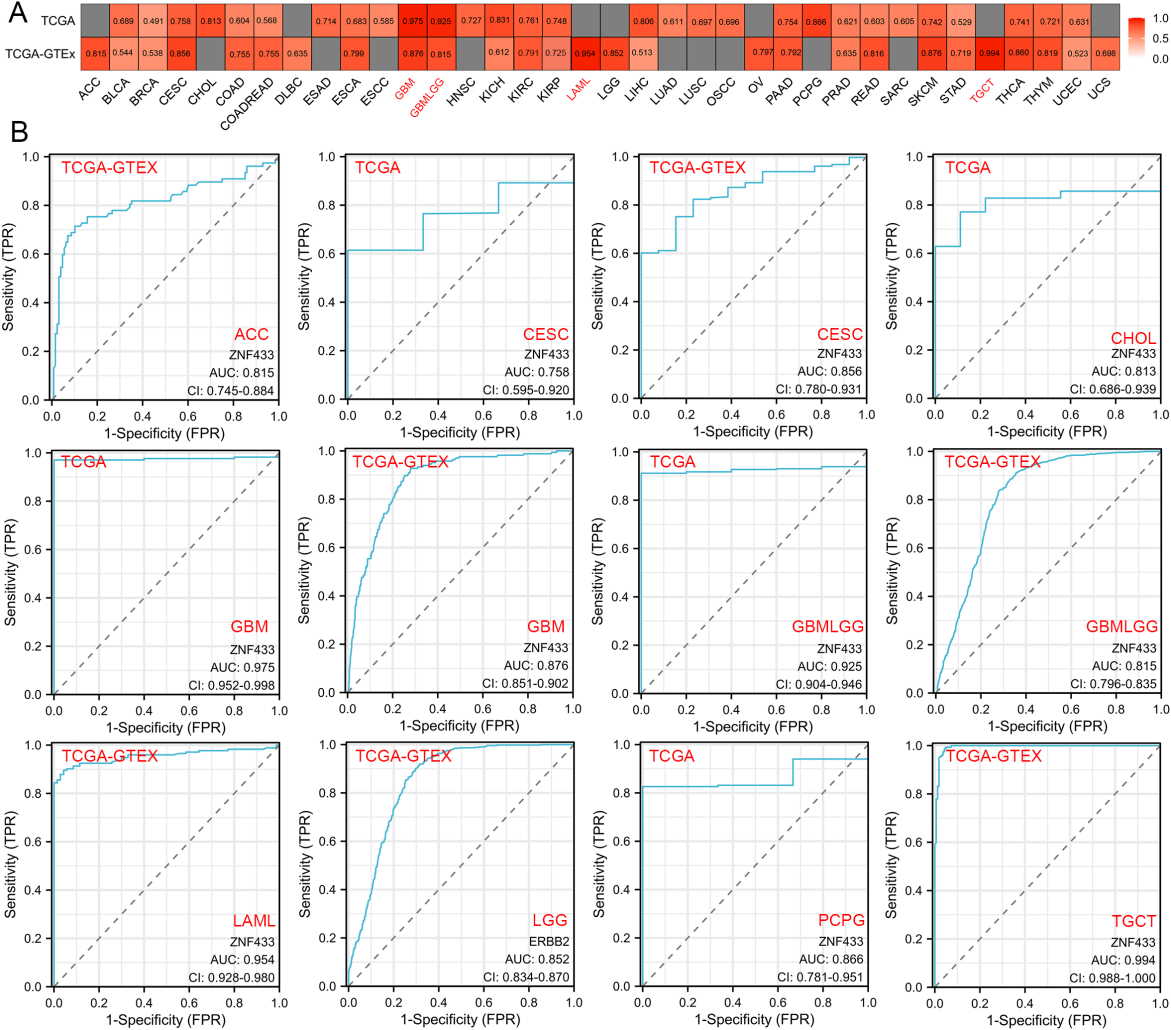
Diagnostic efficiency of the ZNF433 gene in various tumors. **(A)** Heatmap displaying the AUC values of ZNF433 gene expression levels across different cancer types. **(B)** ROC curves for diagnosing specific tumors using the TCGA and TCGA+GTEx datasets for the ZNF433 gene.

Using the TCGA and TCGA+GTEx datasets, we evaluated the diagnostic value of ZNF433 across various cancers (**Figure 9**). The results showed that ZNF433 exhibited significant diagnostic efficacy in several tumors, particularly in ACC, CESC, CHOL, GBM, GBMLGG, KICH, LAML, LGG, LIHC, PCPG, READ, SKCM, TGCT, THCA, and THYM. ROC curve analysis of the TCGA dataset revealed strong diagnostic power for ZNF433 in GBM (AUC = 0.975, 95% CI: 0.952-0.998) and GBMLGG (AUC = 0.925, 95% CI: 0.904-0.946), with AUC values greater than 0.9. For other cancer types such as CESC (AUC = 0.758, 95% CI: 0.595-0.920), CHOL (AUC = 0.813, 95% CI: 0.686-0.939), ESAD (AUC = 0.714, 95% CI: 0.597-0.830), HNSC (AUC = 0.727, 95% CI: 0.664-0.789), KICH (AUC = 0.831, 95% CI: 0.745-0.917), KIRC (AUC = 0.761, 95% CI: 0.710-0.813), KIRP (AUC = 0.748, 95% CI: 0.672-0.824), LIHC (AUC = 0.806, 95% CI: 0.759-0.852), PAAD (AUC = 0.754, 95% CI: 0.434-1.000), PCPG (AUC = 0.866, 95% CI: 0.781-0.951), SKCM (AUC = 0.742, NA-NA), THCA (AUC = 0.741, 95% CI: 0.669-0.813), and THYM (AUC = 0.721, 95% CI: 0.591-0.851), ZNF433 demonstrated moderate diagnostic efficacy.

Using the TCGA+GTEx dataset, we further examined the diagnostic value of ZNF433 across various cancers. ROC curve analysis showed that ZNF433 had strong diagnostic efficacy in LAML (AUC = 0.954, 95% CI: 0.928-0.980) and TGCT (AUC = 0.994, 95% CI: 0.988-1.000), with AUC values exceeding 0.9. Additionally, ZNF433 exhibited moderate diagnostic efficacy in several other cancer types, including ACC, CESC, COAD, COADREAD, ESCA, GBM, GBMLGG, KIRC, KIRP, LGG, OV, PAAD, READ, SKCM, STAD, THCA, and THYM. These findings suggest that ZNF433 may have potential diagnostic value in these cancers. Detailed data can be found in **Supplementary Table 3**.

### 3.9 Drug sensitivity analysis of ZNF433

This study systematically examined the sensitivity of ZNF433 to various drugs, investigating its potential role in modulating cellular drug responses. The results demonstrated that ZNF433 expression was significantly positively correlated with several drugs, including Acetalax (r = 0.427, p = 6.74×10^-4^), Fulvestrant (r = 0.396, p = 1.74×10^-3^), and Bisacodyl (r = 0.359, p = 4.85×10^-3^) (**Supplementary Figure 9**). Conversely, ZNF433 exhibited a significant negative correlation with Trametinib (r = -0.394, p = 1.86×10^-3^), Pralatrexate (r = -0.368, p = 3.78×10^-3^), Cobimetinib (r = -0.343, p = 7.33×10^-3^), and Selumetinib (r = -0.317, p = 1.35×10^-2^) (See **Table 1** for specific data). While most of these drugs are targeted anticancer or chemotherapeutic agents—acting through mechanisms such as hormone receptor modulation, inhibition of the MAPK/ERK signaling pathway, and disruption of DNA synthesis (57–65)—Acetalax and Bisacodyl are primarily used for symptom management (66).

**Table 1:** Drug sensitivity analysis of ZNF433.

Drug	Cor	pvalue
Acetalax	0.426736	0.000674
Fulvestrant	0.39588	0.001742
bisacodyl, active ingredient of viraplex	0.359	0.004849
Arsenic trioxide	0.283919	0.027919
Cordycepin	0.2716	0.0358
Nelarabine	0.268241	0.038245
Raloxifene	0.265311	0.040488
Tanespimycin	-0.26773	0.038632
Ponatinib	-0.27	0.036951
Raltitrexed	-0.27583	0.032907
Selumetinib	-0.31731	0.013498
Cobimetinib (isomer 1)	-0.34284	0.007329
Pralatrexate	-0.36841	0.003776
Trametinib	-0.39367	0.001859

Overall, these drugs share two key characteristics. First, they regulate cellular signal transduction, gene expression, and the cell cycle—processes that are closely linked to cancer initiation and progression. Second, some drugs (e.g., Trametinib (57–59), Cobimetinib (60–62), and Selumetinib (63–65)) specifically target the MEK/ERK signaling pathway. Therefore, ZNF433 may influence cellular proliferation, differentiation, and apoptosis by modulating these pathways, potentially exerting a dual role in anticancer treatment—enhancing the efficacy of certain drugs while attenuating the effects of others. Future studies will focus on elucidating the interactions between ZNF433 and these key signaling pathways, providing a stronger theoretical foundation for the development of personalized anticancer therapies.

### 3.10 Functional pathway enrichment driven by ZNF433 dysregulation

**Figure 10:**
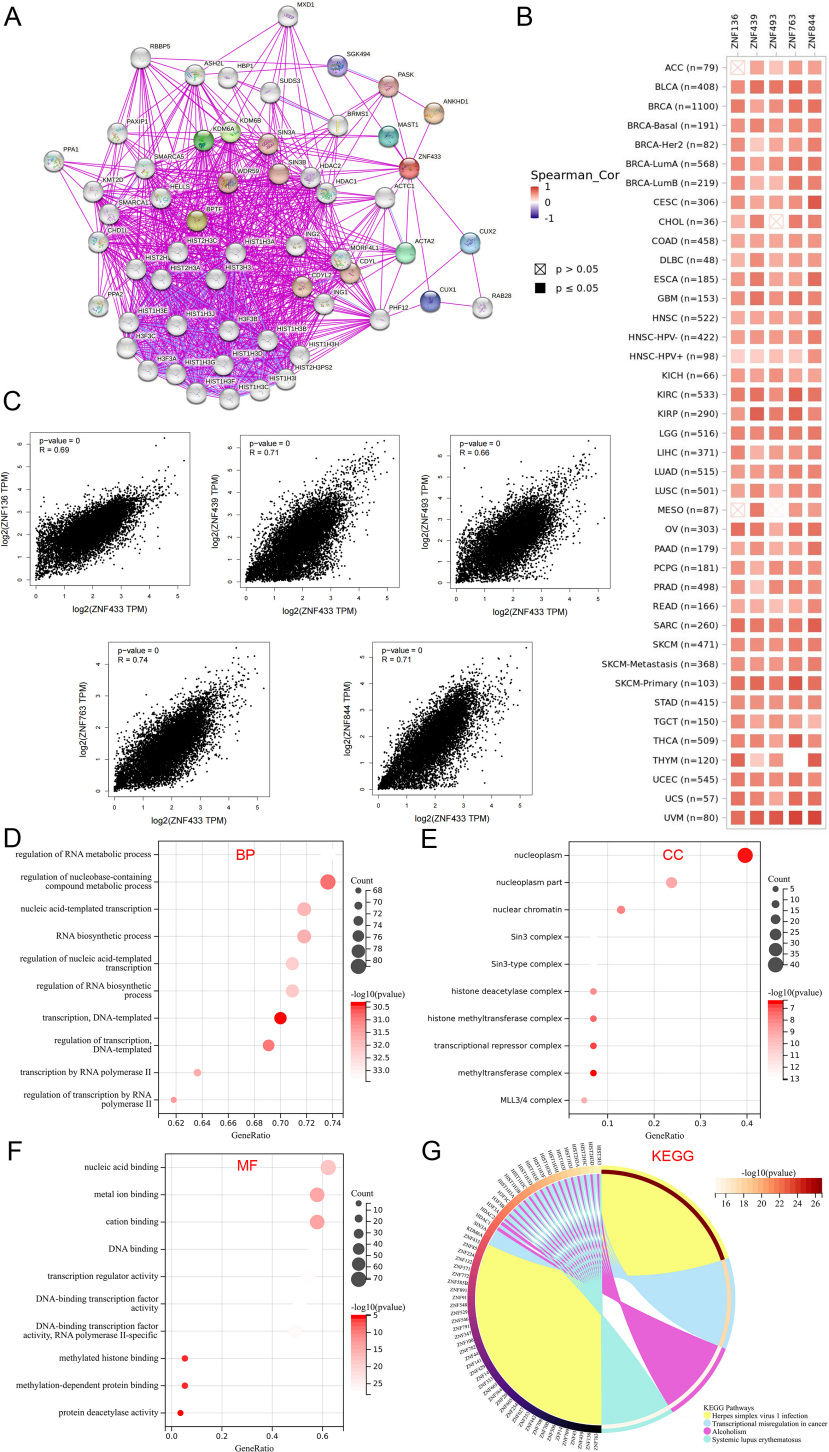
Functional enrichment analysis of ZNF433. **(A)** The STRING database was used to identify proteins interacting with ZNF433. **(B)** The TIMER2 tool was employed to analyze the top 100 genes related to ZNF433 and to display the correlation between ZNF433 and the five most significantly correlated genes (ZNF763, ZNF844, ZNF136, ZNF439, and ZNF493). **(C)** The GEPIA2 tool was used to generate a correlation scatter plot between ZNF433 and the five most correlated genes. **(D-F)** The Sangerbox tool was utilized for GO pathway analysis of ZNF433-related genes and interacting proteins. **(G)** The Sangerbox tool was also used for KEGG enrichment analysis of ZNF433-related genes and interacting proteins.

A comprehensive analysis of protein functional interactions provides critical insights into the mechanisms underlying cancer initiation and progression. In this study, we utilized the STRING database to identify 56 proteins interacting with ZNF433 and constructed an interaction network (**Figure 10A**). Additionally, using GEPIA2, we retrieved the top 100 genes associated with ZNF433 from both tumor and normal tissues in the TCGA database and identified the five most significantly correlated genes. Across all TCGA cancer types, ZNF433 expression exhibited a strong positive correlation with these genes (**Figure 10B**). Specifically, ZNF433 expression was positively correlated with ZNF136 (R = 0.69), ZNF439 (R = 0.71), ZNF493 (R = 0.66), ZNF763 (R = 0.74), and ZNF844 (R = 0.71) (p < 0.001) (**Figure 10C**).

Furthermore, GO and KEGG enrichment analyses were conducted for ZNF433 (**Figures 10D–G**). The GO biological process analysis revealed that ZNF433-related genes and interacting proteins are primarily involved in RNA metabolism regulation, nucleobase-containing compound metabolism regulation, nucleotide-template transcription, RNA biosynthesis, transcription regulation, DNA-templated transcription, RNA polymerase II transcription, and its regulation. Additionally, KEGG enrichment analysis indicated that ZNF433 is primarily associated with pathways such as herpes simplex virus 1 infection, transcriptional dysregulation in cancer, alcoholism, and systemic lupus erythematosus. These findings provide critical insights into the functional characteristics and potential mechanisms of ZNF433 in cancer.

### 3.11 Verification of ZNF433 expression

**Figure 11:**
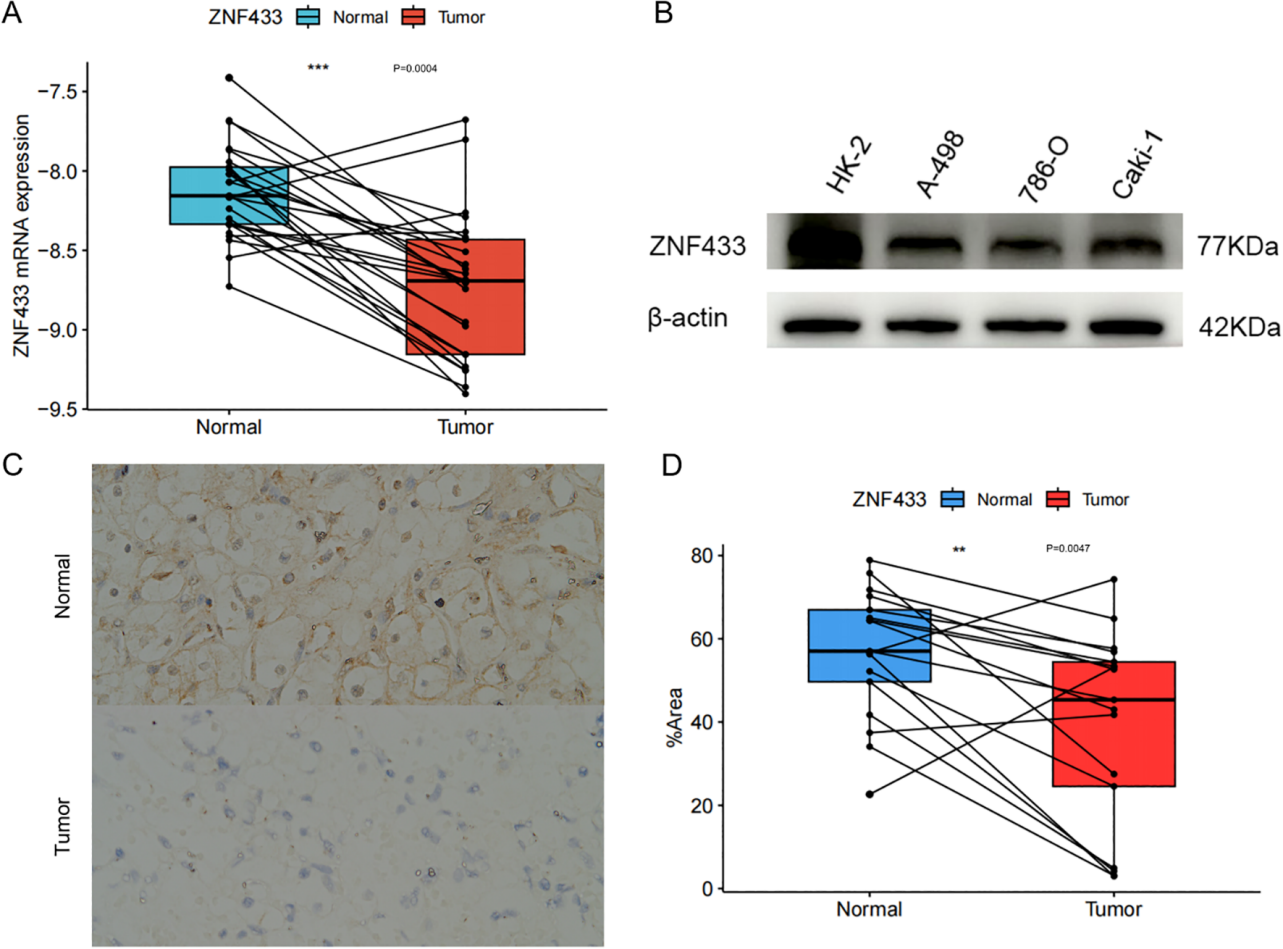
Downregulation of ZNF433 mRNA and protein levels in clear cell renal carcinoma. **(A)** ZNF433 mRNA levels were measured in 25 pairs of clear cell renal carcinoma tissues and paired normal tissues using qPCR. Expression levels were calculated using the ΔCT method, where ΔCT = CT (target gene) - CT (reference gene). **(B)** Western blot analysis was performed to examine ZNF433 protein expression levels in the normal renal tubular epithelial cell line (HK-2) and cancer cell lines. **(C)** Immunohistochemical analysis was conducted on both renal clear cell carcinoma tissues and paired normal tissues. Seventeen cancer tissues and their paired normal tissues were examined, with representative images shown **(C, D)**.

First, the mRNA levels of ZNF433 were examined in 25 ccRCC tissue samples and compared with paired normal kidney tissues. ZNF433 mRNA expression was found to be downregulated in ccRCC tissues (**Figure 11A**). Additionally, the expression of the ZNF433 gene was validated in three renal cancer cell lines (A-498, 786-O, and Caki-1) and one normal kidney cell line (HK-2). The results showed lower levels of ZNF433 protein in renal cancer cells (**Figure 11B**). Furthermore, IHC analysis of 17 cancer tissues and their paired normal counterparts revealed reduced ZNF433 protein levels in the cancer tissues (**Figures 11C, D**). The experimental results demonstrate that both the mRNA and protein levels of ZNF433 are significantly downregulated in ccRCC, which is consistent with the aforementioned bioinformatics analyses.

## 4 Discussion

The KRAB-ZFP family plays a critical role in cancer biology by regulating gene expression, cell cycle progression, and epigenetic modifications, thereby contributing to tumor initiation, progression, and therapeutic response (3, 67–70).Although several KRAB-ZFP family members have been identified as potential diagnostic biomarkers or therapeutic targets (12–15), the functional mechanisms of ZNF433—one of the less well-characterized members—remain poorly understood across various cancer types. This study presents the first comprehensive pan-cancer analysis of ZNF433, integrating multi-dimensional data including gene expression profiles, genetic alterations, prognostic relevance, tumor immune microenvironment, and drug sensitivity to investigate its potential roles in different malignancies.

Analysis results indicate that ZNF433 is significantly downregulated in the majority of cancer types. In both KIRC and KIRP, the expression of ZNF433 progressively decreases with advancing pathological stage, suggesting its potential involvement in tumor progression. This trend implies that ZNF433 may function as a tumor suppressor and could serve as a stage-associated prognostic biomarker in renal cancers.

GO analysis revealed that ZNF433-related genes are primarily involved in RNA metabolism, nucleotide-templated transcription, RNA biosynthesis, and RNA polymerase II-mediated transcriptional regulation, suggesting that ZNF433 may influence cancer cell growth by modulating gene transcription and RNA metabolism. In addition, KEGG pathway analysis indicated that ZNF433 is associated with pathways such as transcriptional dysregulation in cancer, human herpesvirus 1 infection, alcohol addiction, and systemic lupus erythematosus.

Previous studies have shown that ZNF844, a paralog of ZNF433, is also significantly downregulated in KIRC and is associated with poorer survival, advanced pathological stage, and higher tumor grade, suggesting its potential role as a tumor suppressor. ZNF844 is closely related to immune-associated pathways and the tumor microenvironment, and may play a critical role in immune evasion and T cell regulation in ccRCC (13). However, whether ZNF433 shares similar functions with ZNF844 or participates in the same regulatory pathways remains to be elucidated and warrants further investigation.

Survival analysis revealed that ZNF433 has cancer-type-specific prognostic effects: high expression is associated with improved OS in HNSC and KIRC, but with poorer DFS in ESCA and PRAD. Additionally, high ZNF433 expression in BRCA, KIRP, THYM, and KIRC is indicative of better DFS outcomes.

Genetic alteration analysis via cBioPortal identified ZNF433 mutations and amplifications across multiple cancers. Notably, OV patients harboring ZNF433 mutations exhibited significantly reduced OS and DSS, implicating ZNF433 genomic alterations in tumor progression. This finding aligns with the established role of genetic variants in oncogenesis, as exemplified by mutations in KRAS, HER2, and EGFR (71–73). Additionally, it parallels previous observations of JAK3 copy number variations in KIRC (74), ANLN alterations in UCEC (75), and SPDL1 modifications in UCEC outcomes (76).

ZNF433 expression is also closely associated with several genomic instability markers, including TMB, MSI, and MMR. In GBM, SARC, and BLCA, its expression correlates positively with TMB, whereas in BRCA, PAAD, and CHOL, it correlates negatively. These associations suggest that ZNF433 may be involved in DNA repair, maintenance of genomic stability, and epigenetic regulation. Additionally, ZNF433 expression positively correlates with DNA methyltransferases (DNMT1, DNMT3A, DNMT3B) and several RNA modification factors (e.g., METTL14, YTHDF3), indicating its potential role in both transcriptional and post-transcriptional regulation.

Immune microenvironment analysis further revealed tumor-specific immunomodulatory roles. ZNF433 displayed negative correlations with immune regulators and checkpoint molecules in BLCA and GBM, suggesting an immunosuppressive function, while positive associations in DLBC and PRAD implied immune activation. Correlations with tumor-infiltrating lymphocytes across multiple cancers supported ZNF433’s role in microenvironmental regulation.

In terms of diagnostic value, ROC analysis demonstrated that ZNF433 exhibits strong diagnostic potential in LAML and TGCT (AUC > 0.9), with moderate diagnostic efficacy in other cancers. Drug sensitivity analysis based on CellMiner data revealed significant correlations between ZNF433 expression and response to various anticancer agents. Notably, high ZNF433 expression was associated with reduced sensitivity to MEK inhibitors (e.g., Trametinib, Cobimetinib, Selumetinib), suggesting that ZNF433 may enhance treatment resistance by modulating the MAPK/ERK pathway, highlighting its potential as a predictive biomarker for targeted therapy response.

Despite these novel findings, several limitations must be acknowledged. First, our study primarily relies on bioinformatics analyses based on public databases, and the specific roles of ZNF433 in RNA modification, immune regulation, and drug resistance mechanisms remain unvalidated experimentally. Second, immune infiltration correlations were based on database analysis and still require orthogonal validation through techniques such as multiplex immunohistochemistry or spatial transcriptomics. Lastly, the correlations between ZNF433 and drug sensitivity need to be further validated in preclinical models, including cellular and animal studies, to confirm its therapeutic potential.

In conclusion, this study provides the first multidimensional characterization of ZNF433 across various cancers, delineating its potential in diagnostic, prognostic, and personalized treatment applications. These findings not only expand our understanding of KRAB-ZFP-mediated tumor regulation but also provide a theoretical foundation and research framework for future studies on ZNF433-driven mechanisms and clinical translational applications.

## 5 Conclusions

This study presents the first comprehensive pan-cancer analysis of ZNF433, revealing its promising potential as a diagnostic and prognostic biomarker. Our findings provide new insights into the potential of ZNF433 as a cancer biomarker and therapeutic target, laying the foundation for future mechanistic studies and clinical applications.

## Data Availability

The original contributions presented in the study are included in the article/**Supplementary Material**. Further inquiries can be directed to the corresponding author.
