# Supplementary material for: Multidimensional analysis suggests that ZNF433 is a promising biomarker for the diagnosis and prognosis of human cancers
Source: Front Oncol. 2025 Jun 25;15:1584042. doi: 10.3389/fonc.2025.1584042 (PMC12237650; doi:10.3389/fonc.2025.1584042)
Supplement: Supplementary file 1 [file DataSheet1.docx]

Supplementary Material

# Supplementary Figures and Tables

## Supplementary Figures


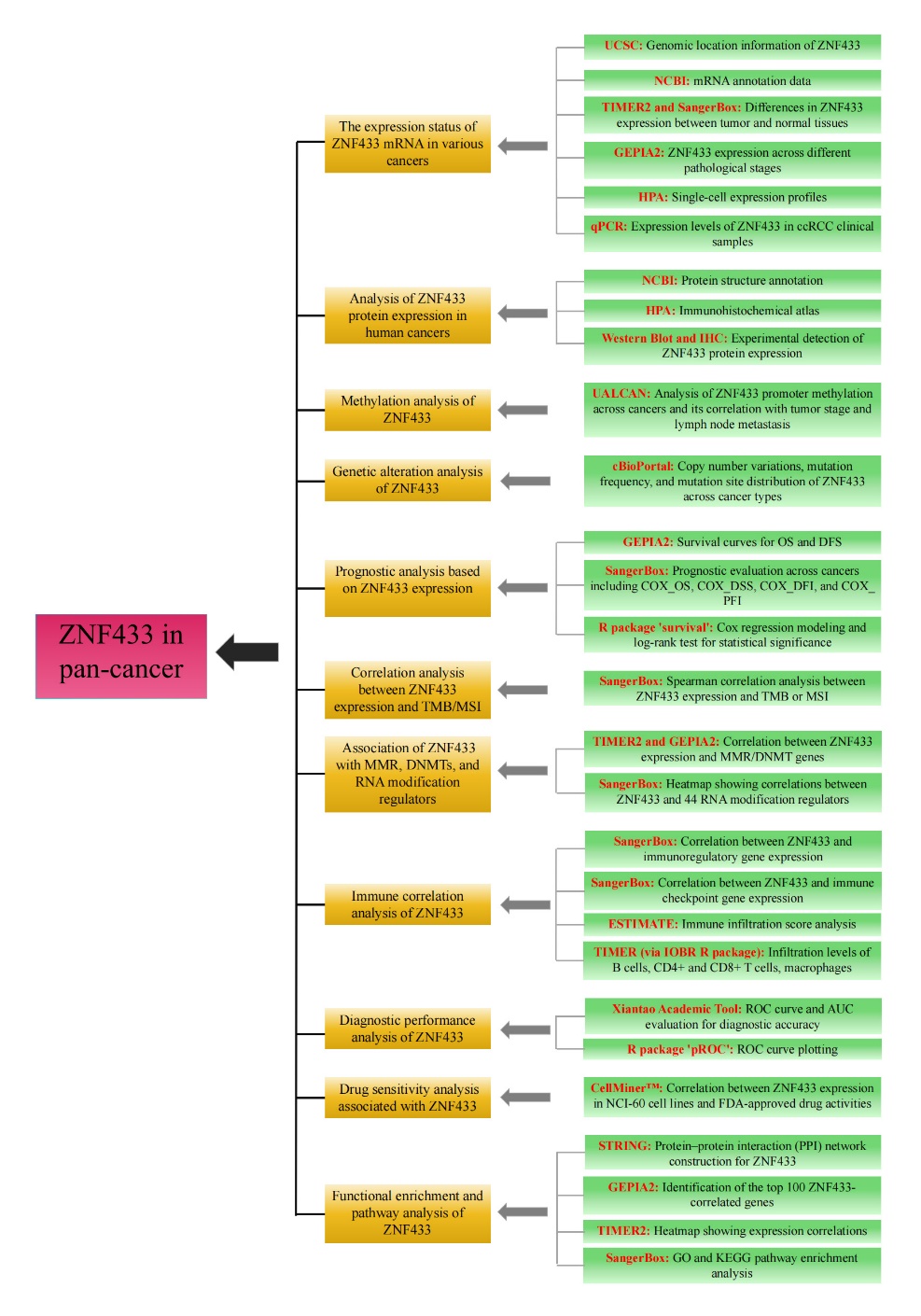


**Supplementary Figure 1.** Multidimensional Bioinformatics Analysis Workflow of ZNF433 in Pan-Cancer.


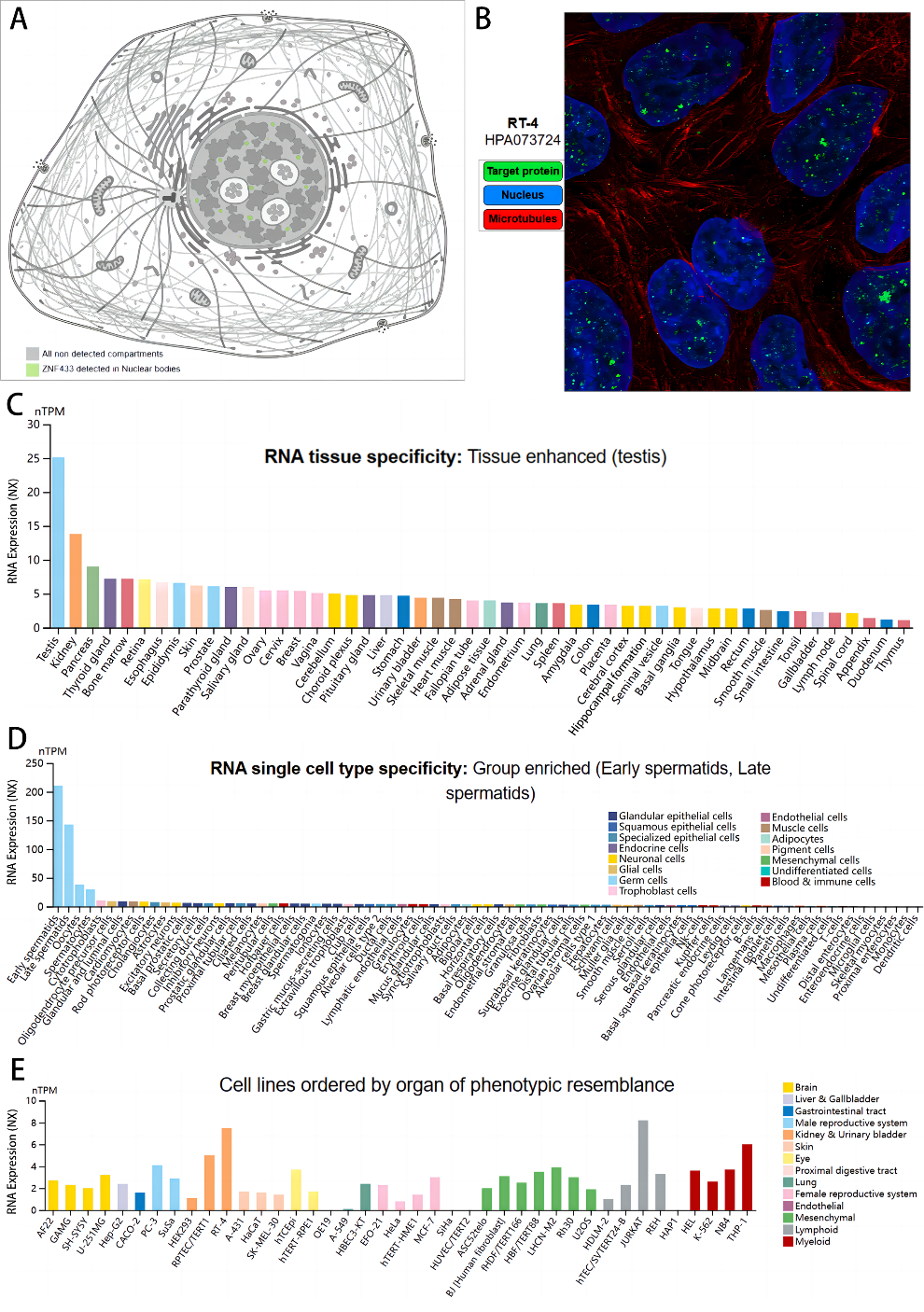


**Supplementary Figure 2.** Landscape of ZNF433 subcellular localization and expression profiles across various tissues, single-cell types, and cell lines.(A) ZNF433 subcellular localization in the nucleolus.(B) Immunofluorescence staining of the human RT-4 cell line showing nuclear localization of ZNF433.(C) Normalized eXpression (NX) levels of ZNF433 across 55 normal tissue types and 6 blood cell types, derived from three transcriptomic datasets (HPA, GTEx, and FANTOM5).(D) Expression levels of ZNF433 in various single-cell types.(E) Expression levels of ZNF433 in different cell lines.


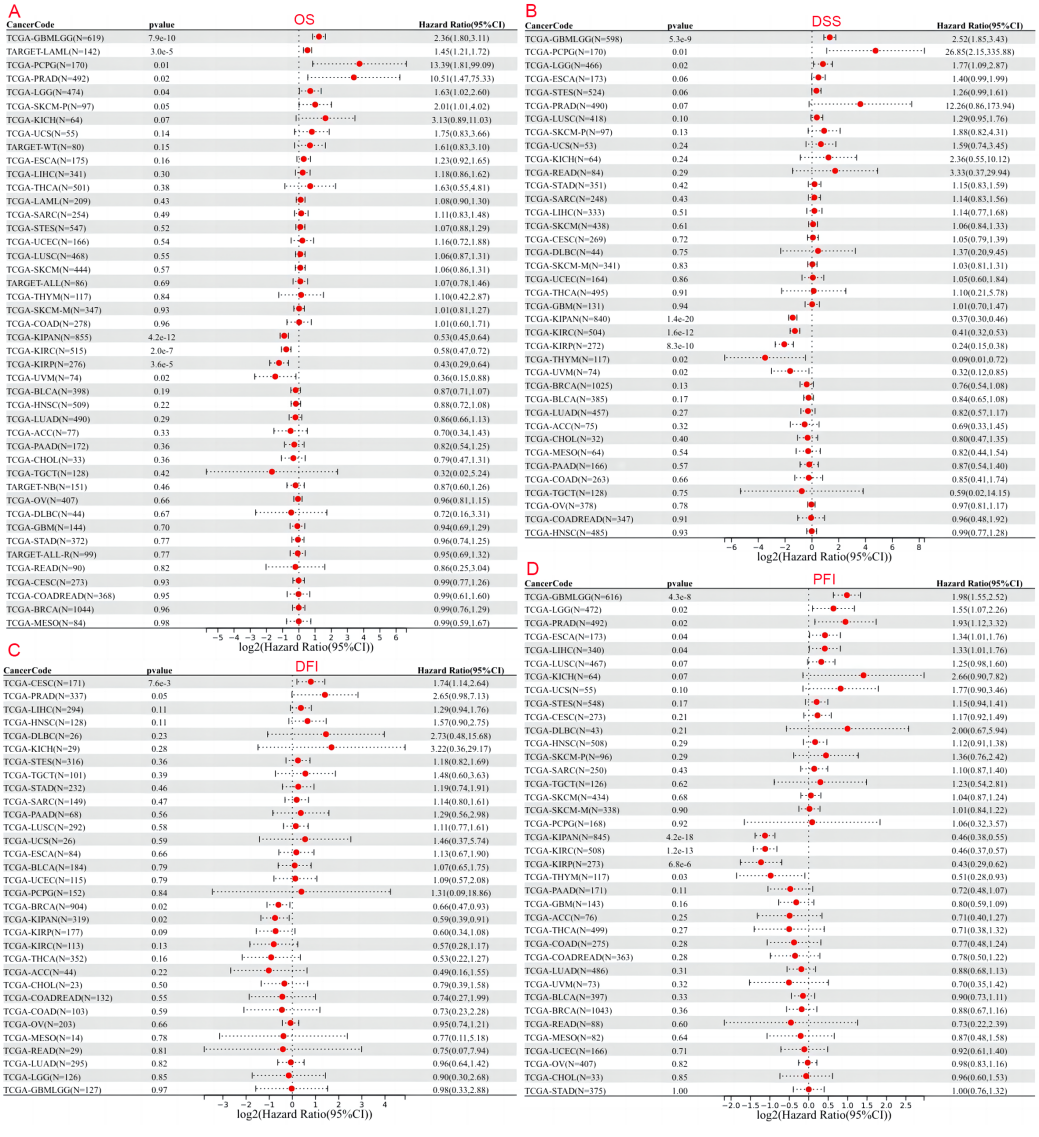


**Supplementary Figure 3**. Forest plots of univariate Cox regression analyses for OS (A), DSS (B), DFI (C), and PFI (D).


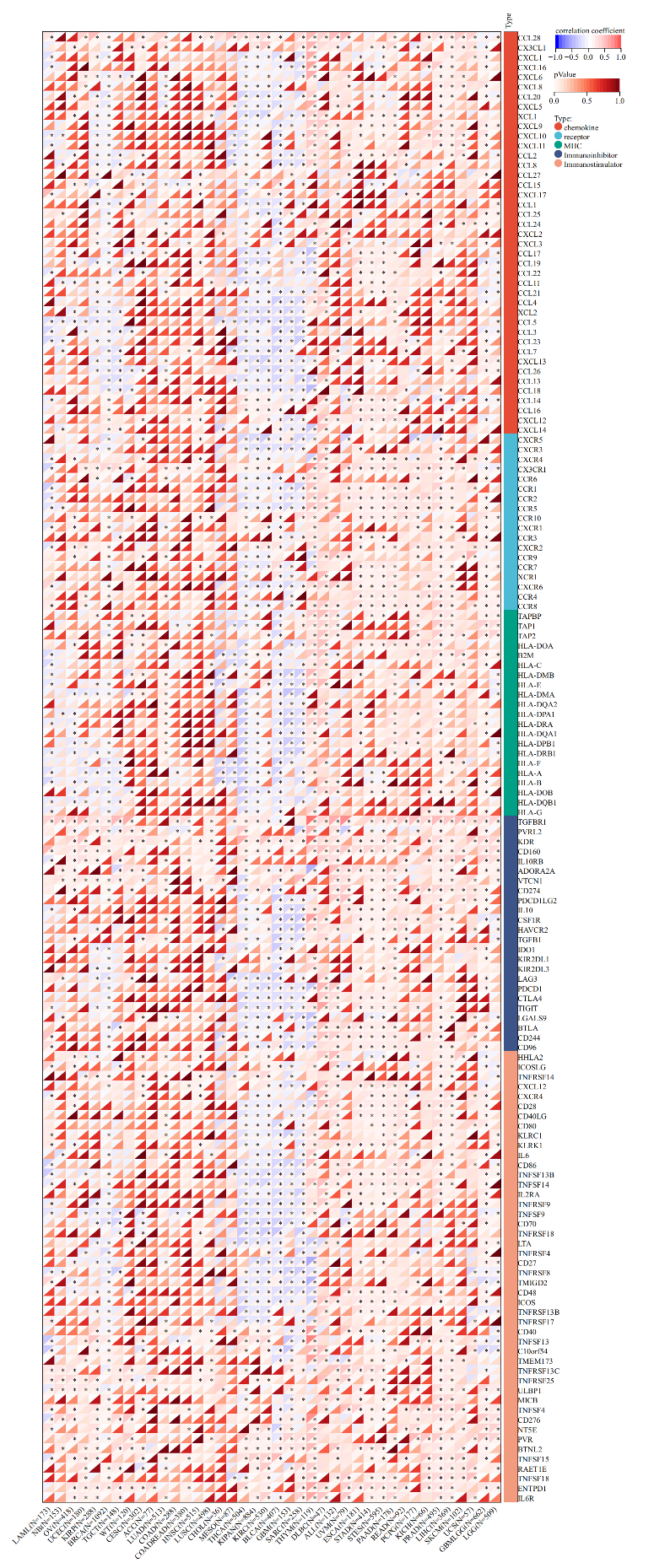


**Supplementary Figure 4.** Correlation between ZNF433 and 150 immunoregulatory genes (chemokines, receptors, MHC, and immune stimulants).


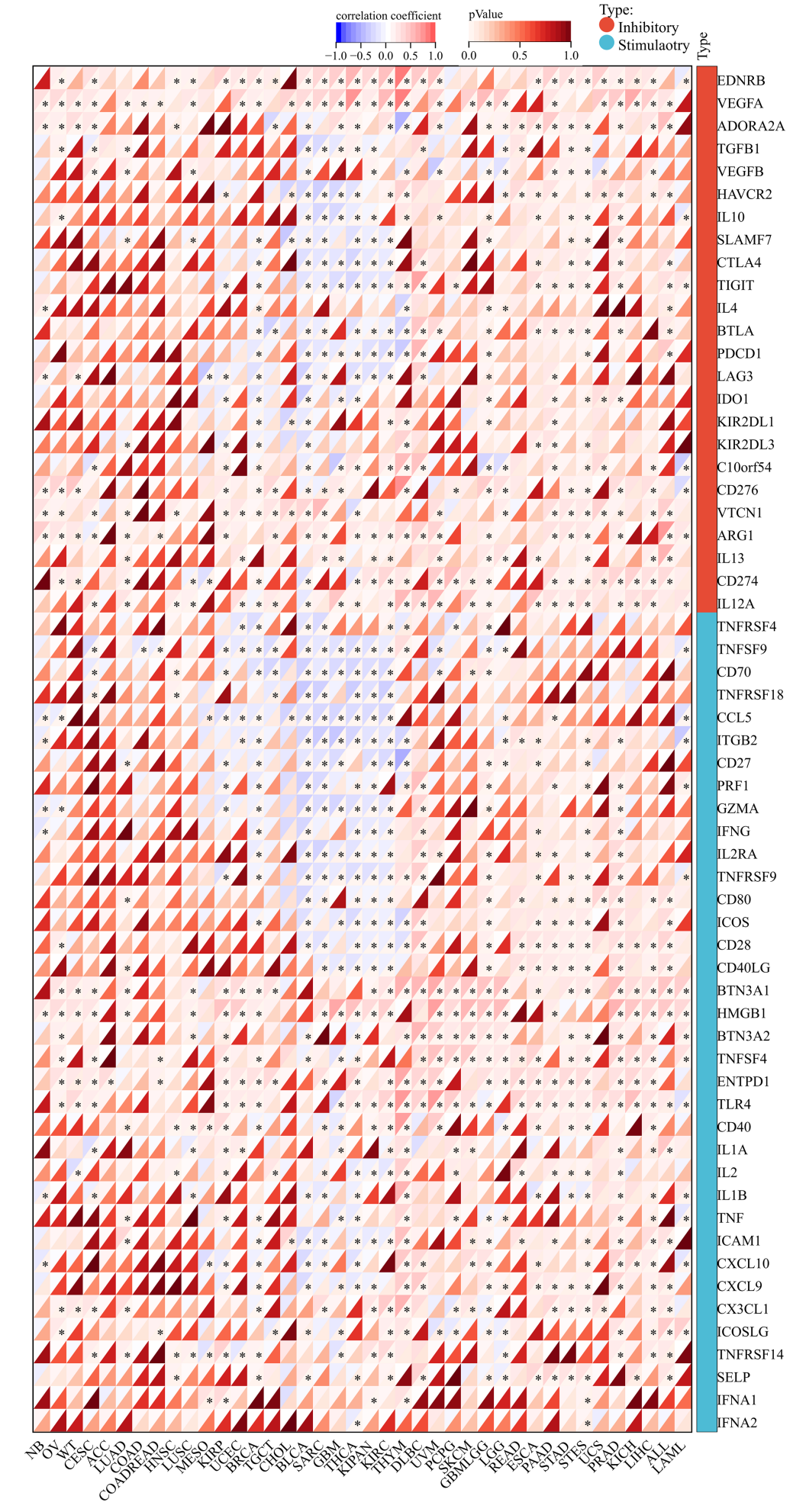


**Supplementary Figure 5.** Correlation between ZNF433 and 60 immune checkpoint genes (24 inhibitory and 36 stimulatory genes).


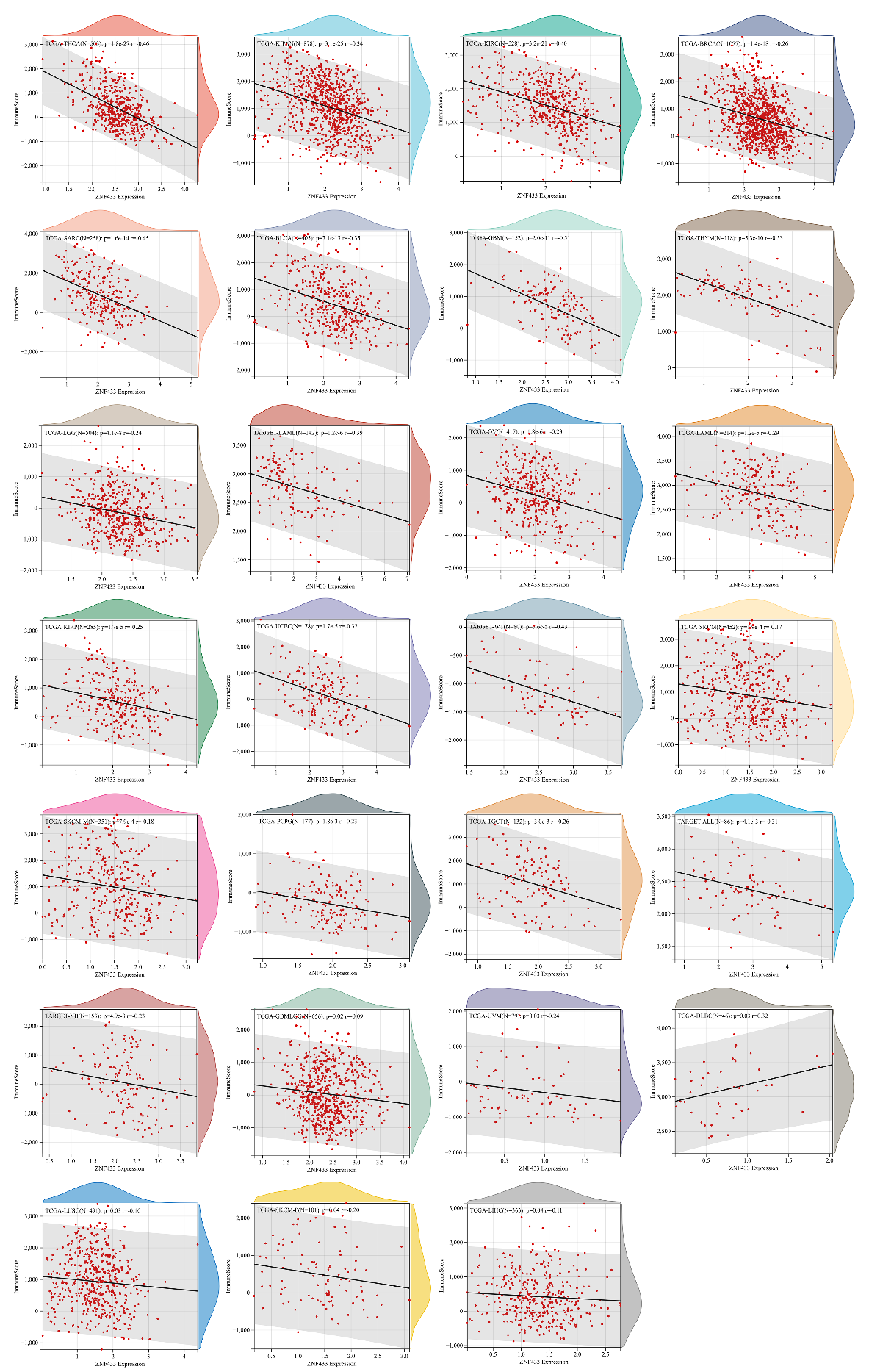


**Supplementary Figure 6.** Correlation between ZNF433 gene expression and immune cell infiltration across 27 cancer types.


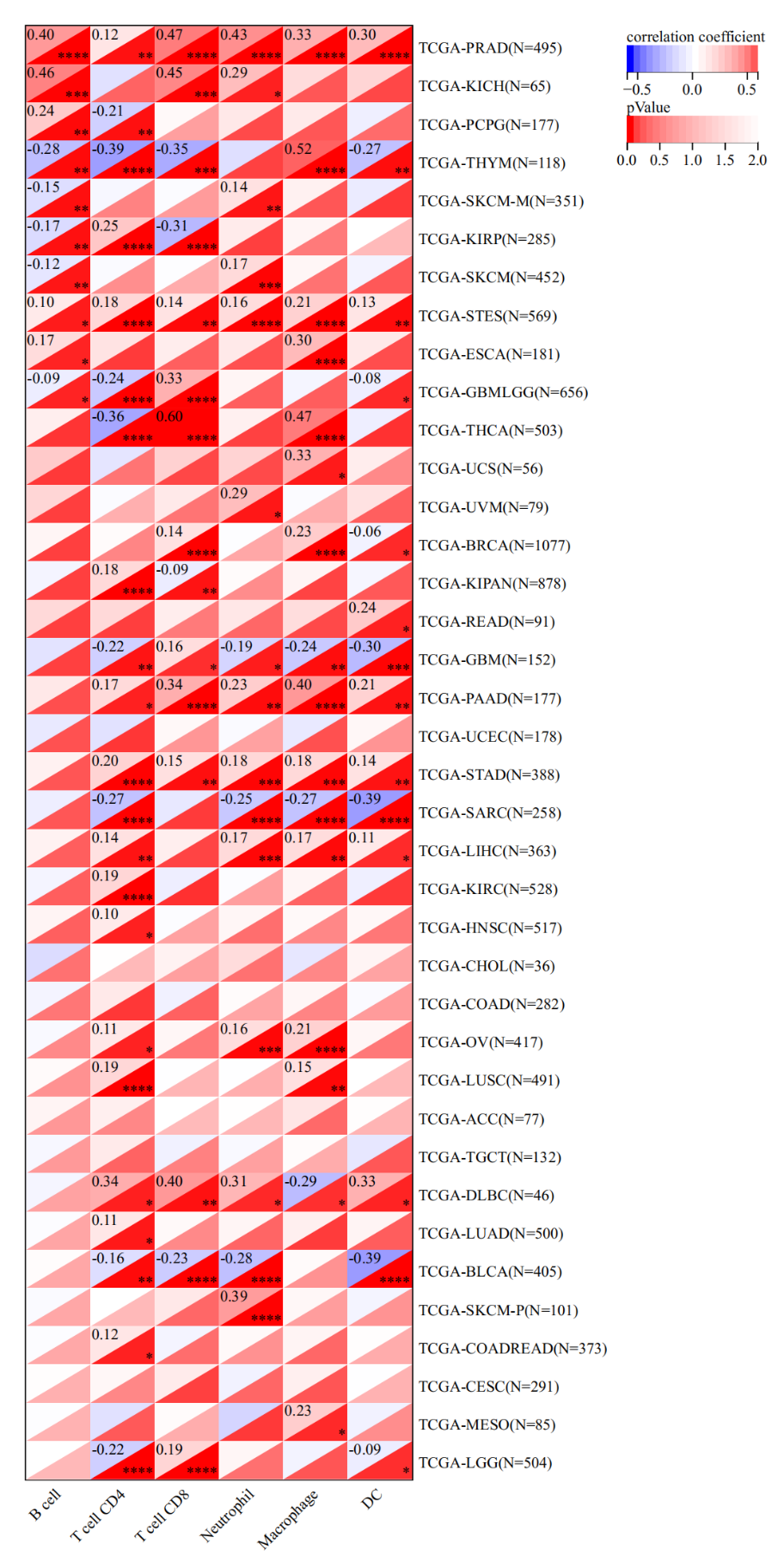


**Supplementary Figure 7.** Correlation between ZNF433 gene expression and infiltration scores of six immune cell types.


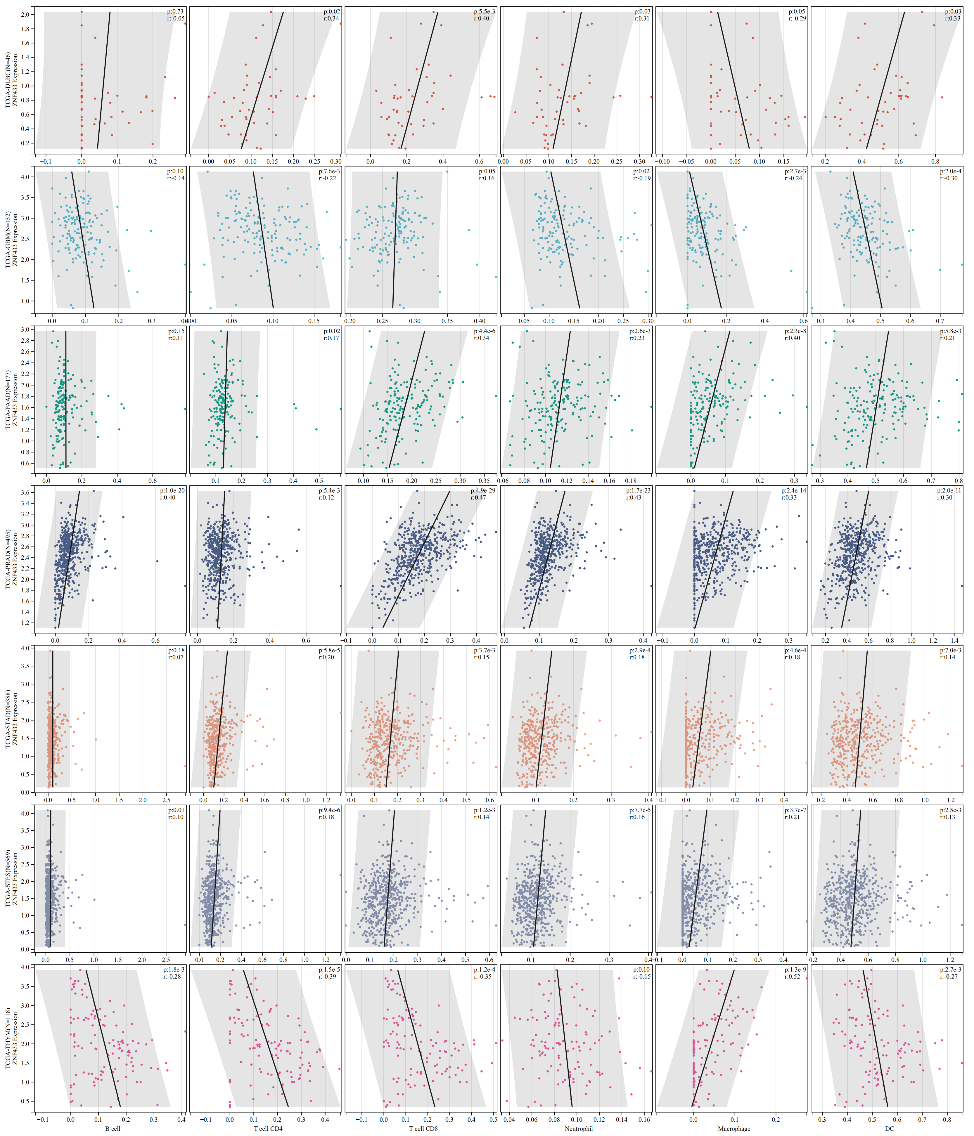


**Supplementary Figure 8.** Association between ZNF433 expression and infiltration scores of the six immune cell types in the six tumors most strongly correlated with ZNF433.


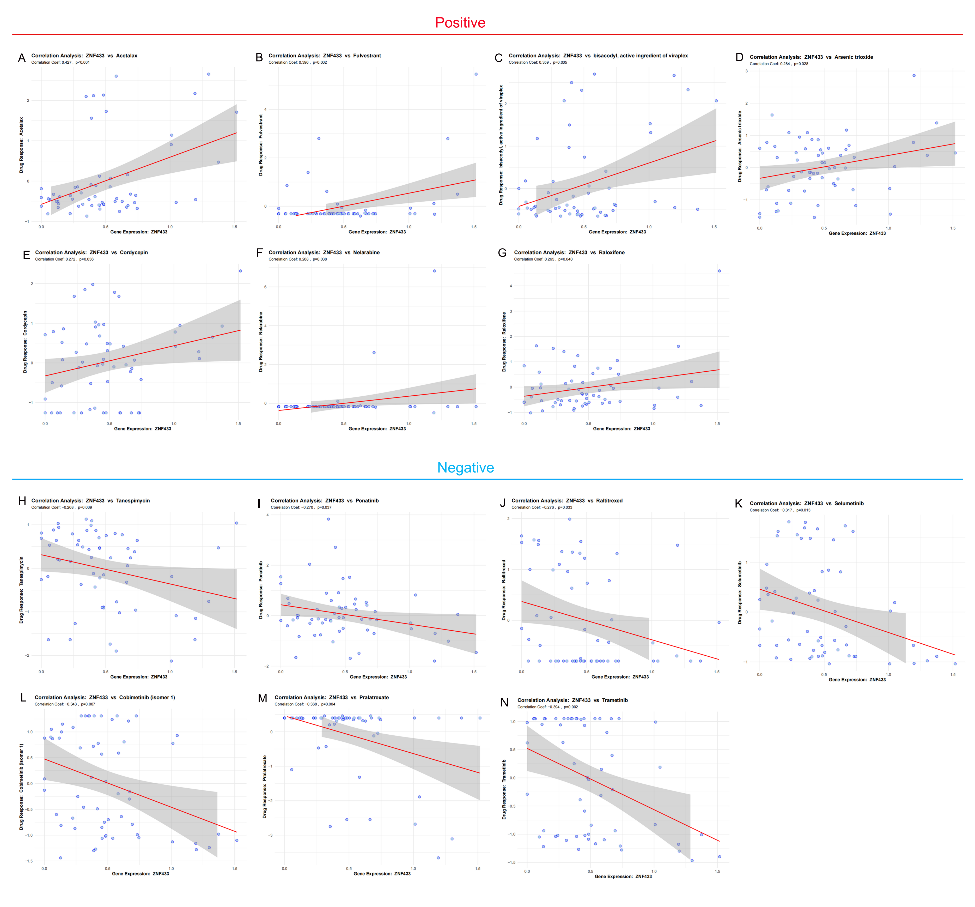


**Supplementary Figure 9.** Drug Sensitivity Analysis of ZNF433.The expression of ZNF433 was significantly correlated with the sensitivity of multiple drugs, including Acetalax (A), Fulvestrant (B), and Bisacodyl, the active ingredient of Viraplex (C). Additionally, significant correlations were observed with Arsenic Trioxide (D), Cordycepin (E), Nelarabine (F), Raloxifene (G), Tanespimycin (H), Ponatinib (I), Raltitrexed (J), Selumetinib (K), Cobimetinib (Isomer 1) (L), Pralatrexate (M), and Trametinib (N).
